# Identification of the prokaryotic ligand-gated ion channels and their implications for the mechanisms and origins of animal Cys-loop ion channels

**DOI:** 10.1186/gb-2004-6-1-r4

**Published:** 2004-12-20

**Authors:** Asba Tasneem, Lakshminarayan M Iyer, Eric Jakobsson, L Aravind

**Affiliations:** 1 Beckman Institute, University of Illinois at Urbana-Champaign, 405 N Mathews Avenue, Urbana, IL 61801, USA; 2 National Center for Biotechnology Information, National Library of Medicine, National Institutes of Health, Bethesda, MD 20894, USA

## Abstract

**Background:**

Acetylcholine receptor type ligand-gated ion channels (ART-LGIC; also known as Cys-loop receptors) are a superfamily of proteins that include the receptors for major neurotransmitters such as acetylcholine, serotonin, glycine, GABA, glutamate and histamine, and for Zn^2+ ^ions. They play a central role in fast synaptic signaling in animal nervous systems and so far have not been found outside of the Metazoa.

**Results:**

Using sensitive sequence-profile searches we have identified homologs of ART-LGICs in several bacteria and a single archaeal genus, *Methanosarcina*. The homology between the animal receptors and the prokaryotic homologs spans the entire length of the former, including both the ligand-binding and channel-forming transmembrane domains. A sequence-structure analysis using the structure of *Lymnaea stagnalis *acetylcholine-binding protein and the newly detected prokaryotic versions indicates the presence of at least one aromatic residue in the ligand-binding boxes of almost all representatives of the superfamily. Investigation of the domain architectures of the bacterial forms shows that they may often show fusions with other small-molecule-binding domains, such as the periplasmic binding protein superfamily I (PBP-I), Cache and MCP-N domains. Some of the bacterial forms also occur in predicted operons with the genes of the PBP-II superfamily and the Cache domains. Analysis of phyletic patterns suggests that the ART-LGICs are currently absent in all other eukaryotic lineages except animals. Moreover, phylogenetic analysis and conserved sequence motifs also suggest that a subset of the bacterial forms is closer to the metazoan forms.

**Conclusions:**

From the information from the bacterial forms we infer that cation-pi or hydrophobic interactions with the ligand are likely to be a pervasive feature of the entire superfamily, even though the individual residues involved in the process may vary. The conservation pattern in the channel-forming transmembrane domains also suggests similar channel-gating mechanisms in the prokaryotic versions. From the distribution of charged residues in the prokaryotic M2 transmembrane segments, we expect that there will be examples of both cation and anion selectivity within the prokaryotic members. Contextual connections suggest that the prokaryotic forms may function as chemotactic receptors for low molecular weight solutes. The phyletic patterns and phylogenetic relationships suggest the possibility that the metazoan receptors emerged through an early lateral transfer from a prokaryotic source, before the divergence of extant metazoan lineages.

## Abstract 2

Acetylcholine receptor type ligand-gated ion channels are well known in animals. Homologs are identified in prokaryotes that may act as chemotactic receptors.

## Background

The flux of ions across excitable cellular membranes is a signaling mechanism that is extensively utilized by organisms from all the three major superkingdoms of life. This directional flow of ions across cellular membranes is mediated by a wide range of ion channels that may be gated by a variety of signals, such as voltage, mechanical forces or chemical first messengers [1]. Ion-dependent signaling is particularly critical for the functions of the animal nervous system, where propagation of signals along neuronal processes and the transmission of signals from neurons or receptor cells to their targets is mediated by the action of ion channels. The neuronal ligand- or neurotransmitter-gated ion channels (LGICs) combine the functionalities of a receptor and ion channel in a single protein, and mediate fast synaptic signaling [1]. The neurotransmitter released by the presynaptic cell, within a few microseconds binds to the extracellular ligand-binding module of the ion channel and causes the channel to open. This results in a selective flow of ions down their electrochemical gradients through the water-filled pore of the channel, and the excitation or inhibition of the train of action potentials in the postsynaptic cells. Furthermore, within a few milliseconds the neurotransmitter dissociates from the receptor and thereby terminates the synaptic signal. Thus, the LGICs act as molecular switches to provide a specific impulse of ion flux in response to a neuronal signal [1]. One of the most prominent superfamilies of the animal LGICs has as its prototype the acetylcholine-gated channels and includes the receptors for a variety of neurotransmitters in both vertebrates and invertebrates ([2], also see [3]). The known endogenous ligands bound by these receptors are acetylcholine, GABA, serotonin, glycine, histidine, glutamate and cationic zinc [4-8]. The receptors are also the targets of plant toxins such as nicotine and strychnine, conotoxins of snails, lophotoxins of corals, and many of the neurotoxins of elapid snakes [4-6]. This superfamily is commonly referred to as the Cys-loop superfamily (named after a conserved cystine bridge seen in the animal representatives of this superfamily) or the acetylcholine-receptor-type LGIC superfamily (ART-LGIC).

All the known members of this superfamily possess stereotypic domain architectures, with an all-β amino-terminal ligand-binding domain (LBD) and a carboxy-terminal transmembrane domain comprised of four membrane-spanning helices (4-TM). The members of this superfamily exhibit a pentameric quaternary structure, with the second transmembrane helix from each monomer (helix M2) contributing to the wall of a transmembrane pore through which the ion passes. The animal ART-LGICs may exist as heteropentamers, containing up to four distinct paralogous monomers. The ligand is bound at the dimer interface of two adjacent LBDs, and residues from both subunits form a box-like cavity to accommodate the ligand [9,10]. In the case of most animal neurotransmitter receptors in their open state, only two (or occasionally three) of the five subunit junctions in the pentameric receptor are occupied by the ligand [4-6].

The ART-LGICs characterized to date show ion selectivity. The excitatory channels, such as the acetylcholine and serotonin receptors, the mammalian Zn receptors and some invertebrate GABA receptors, allow the flow of cations, whereas the inhibitory receptors, such as those for glycine and GABA, invertebrate glutamate and histamine receptors, and some invertebrate serotonin receptors (such as *Caenorhabditis elegans *MOD-1), allow the flow of anions. Cation or anion selectivity of the channel is principally governed by the charge distribution in the linker between the transmembrane helices M1 and M2 [11,12].

Several recent studies based on the X-ray structure of the recombinant homopentamer of the soluble acetylcholine-binding domain (ACHB) from the snail *Lymnaea stagnalis *[9] and the electron microscopic structure of the transmembrane domain [13] have thrown light on the possible mechanisms of ligand interaction and channel gating of the ART-LGICs. The current model for the mechanism of these channels posits that the binding of the ligand causes a preferential rotation of one of the β sheets of the LBD. The resultant conformational change is believed to be transmitted via interactions with the loop between helices M2-M3 to the hydrophobic constriction in the middle of the M2 helices that line the channel walls [13]. This causes a relaxation of the middle of the girdle and allows the flow of the ions. Despite intense studies, there remain several unresolved issues with respect to the mechanism by which the binding of the ligand to a segregated site transmits the conformational change to the rest of the LBDs to trigger the rotation. Furthermore, the extent of the applicability of the conclusions drawn from the acetylcholine receptor model for other members of the superfamily remains somewhat unclear.

Thus far, the ART-LGIC superfamily is known only from multicellular animals (metazoans). Phylogenetic analysis suggests that the common ancestor of the bilateral animals already possessed multiple members belonging to two major families of the superfamily that correspond, respectively, to the excitatory cationic channels, including the acetylcholine and serotonin receptors, and the inhibitory anionic channels, including the GABA, glycine and invertebrate histamine and glutamate receptors [2,14,15]. This restricted phyletic pattern is in contrast with what has been previously observed for the voltage-gated potassium channels of the Shaker-type superfamily and the voltage-gated sodium channels. In both these cases, several representatives are known from both non-animal eukaryotes, as well as numerous prokaryotes, suggesting that they were employed in signaling in other contexts well before the origin of the animal nervous system [16-19]. This prompted us to investigate if distant representatives of the Cys-loop/ART-LGIC superfamily could be detected in organisms outside the animal lineage. We also sought to use these distant relatives in comparative sequence-structure and genomics studies to understand the most general functional and mechanistic features that typify this superfamily.

We report here the identification of several prokaryotic members of the ART-LGIC superfamily and discuss the general implications of these proteins for the mechanisms and origin of the Cys-loop receptors of the animal nervous system.

## Results and discussion

### Identification of prokaryotic versions of the ART-LGIC superfamily

To investigate the origins of the animal ART-LGIC superfamily, we tried to obtain a complete picture of their phyletic spread in all organisms with currently available genomic sequence information. All *bona fide *animal members of this superfamily (with the exception of snail ACHB) contain a globular, extracellular, amino-terminal LBD and a carboxy-terminal 4-TM domain. The membrane-spanning helices, being compositionally biased, tend to frequently recover false positives in iterative sequence profile searches. Accordingly, we only used the globular extracellular domains of the known ART-LGIC receptors, which are typically around 200-220 amino acids in length, for our iterative sequence profile searches with the PSI-BLAST program.

Iterative searches from a number of starting queries, such as the human acetylcholine receptor α7 chain (gi: 2144875; region 24-230), *C. elegans *MOD-1 receptor (gi: 25154135; region 32-238) or the human GABA receptor α4 chain (gi: 1346079; 46-256) recovered a consistent set of receptors from diverse animals with significant expect (e)-values prior to convergence (run with inclusion threshold of 0.01). Interestingly, in addition to the animal sequences these searches also recovered sequences from different bacteria. For example a search initiated with the above-mentioned acetylcholine receptors as the seed recovered *Gloeobacter violaceus*, *Crocosphaera watsonii *(both cyanobacteria) in iteration 3 (e-values = 10^-5^-10^-7^) and *Rhodopseudomonas palustris *(α-proteobacteria) in iteration 6 (e-value = 10^-4^). However, no significant hits belonging to any of the other eukaryotic lineages, such as the fungi, *Dictyostelium*, plants, alveolates or *Giardia *were detectable. To further investigate the occurrence of ART-LGIC homologs in bacteria, we constructed a PSI-BLAST profile of the LBDs recovered in the above searches and used it to systematically search all the bacterial genomes, which are available as whole-genome shotgun reads or as completely assembled chromosomes. As a result of these searches we recovered statistically significant hits to the ART-LGIC LBDs from several other phylogenetically diverse bacteria including *Cytophaga hutchinsonii*, α-proteobacteria like *Bradyrhizobium japonicum *and *Magnetospirillum magnetotaticum*, γ-proteobacteria, like *Erwinia chrysanthemi*, *Microbulbifer degradans *and *Methylococcus capsulatus*, several cyanobacteria and a single archaeal genus *Methanosarcina*.

All these bacterial hits corresponded to the full length of the animal LBDs, which were used as seeds to build the sequence profiles. When signal peptides and the transmembrane helices were predicted for the bacterial proteins, all of them showed a general structure similar to the animal receptors; that is, an amino-terminal signal peptide and a LBD followed by a carboxy-terminal 4-TM domain. However, some of the bacterial proteins showed additional domains between the amino-terminal signal peptide and the ART-LGIC superfamily ligand-binding and channel domains (see below for further discussion). Reciprocal searches with either just the region corresponding to the LBD or the whole unit comprising both the LBD and the following 4-TM domain of the bacterial proteins recovered the animal Cys-loop proteins with significant e-values (0.001-10^-17 ^in iterations 1-3). For example, a search with the sequence of Chut0841 (gi: 23135736) from *C. hutchinsonii *recovered the animal receptors with e-values in the range 10^-4^-10^-6 ^in the second iteration. The secondary structure was predicted for the region corresponding to the LBD in the bacterial proteins using the programs PHD [20] and JPRED2, using the combined information from the multiple alignment, a PSI-BLAST position-specific score matrix and a hidden Markov model derived from the alignment [21]. The predicted secondary structure of the bacterial proteins precisely corresponded to the secondary structure of the conserved core of the animal LBDs typified by the ACHB (PDB:1UV6), with an amino-terminal helix followed by nine β strands, which form a β sandwich [9].

Taken together, the above observations suggested that the bacterial proteins were *bona fide *homologs of the animal neurotransmitter receptors of the ART-LGIC/Cys-loop superfamily.

### Mechanistic and functional implications of the comparative sequence-structure analysis of the bacterial and animal ART-LGIC receptors

**Figure 1:**
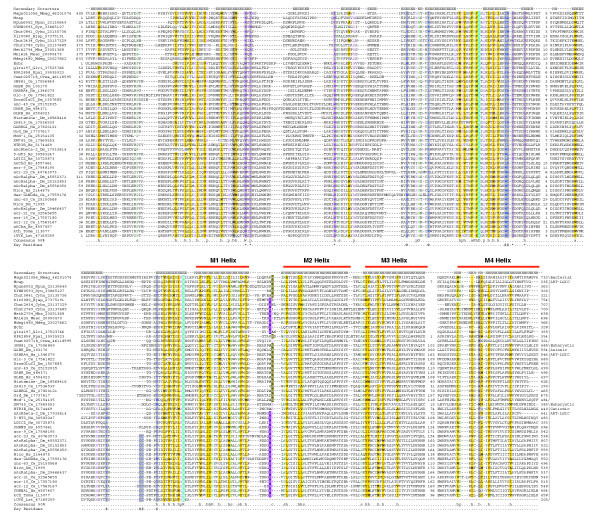
A multiple alignment of the ART-LGIC/Cys-loop superfamily (see also Additional data file 2 for alignment; an alignment of metazoan members only may also be obtained from PFAM: PF02931 LBDs; PF02932: TM domain). Proteins are denoted by their gene names, species abbreviations and gi. The secondary-structure assignments, based on the available crystal structures of the acetylcholine receptor pore (pdb: 1OED) and Achbp protein (pdb: 1UV6), are shown above the alignment where E denotes extended or strand, and H, helix. The coloring reflects the composition of the amino acids at 90% consensus. The coloring scheme and the consensus abbreviations are as follows: h, hydrophobic (ACFILMVWY), l, aliphatic residues (ILV), and a, aromatic residues (FHWY) are shaded yellow; s, small (AGSVCDNPT) and u, tiny residues (GAS) are colored green; c, charged (DEHKR), +, basic (HKR), -, acidic (DE), p, polar (CDEHKNQRST) are colored magenta. The conservation pattern as plotted onto the three-dimensional structure of the ACHB is shown in Additional data file 1. Also shown below the alignment are the key residues described in the text. # and @ represent residues of adjacent chains (PDB id: 1UV6, chain C and chain D respectively) involved in ligand binding (shaded gray). Residues predicted to be potentially involved in the transmission of conformational change are marked by an asterisk (*) at the bottom of the column and are colored violet and shaded gray. The highly conserved positions - the acidic residue in the middle of the Cys-loop and the basic residue at the carboxyl terminus of the LBD - are shown in inverse blue shading. The arginine residue involved in ion selectivity in anionic channels is shaded green and the glutamate residue involved in ion selectivity in cationic channels is shaded purple. Species abbreviations are as follows: Bjap, *Bradyrhizobium japonicum*; Ce, *Caenorhabditis elegans*; Crwa, *Crocosphaera watsonii*; Cyhu, *Cytophaga hutchinsonii*; Dm, *Drosophila melanogaster*; Echr, *Erwinia chrysanthemi*; Glvi, *Gloeobacter violaceus*; Hs, *Homo sapiens*; Lst, *Lymnaea stagnalis*; Mba, *Methanosarcina barkeri*; Mcap, *Methylococcus capsulatus*; Mdeg, *Microbulbifer degradans*; Meac, *Methanosarcina acetivorans*; Mmag, *Magnetospirillum magnetotacticum*; Npun, *Nostoc punctiforme*; Rpal, *Rhodopseudomonas palustris*; Syn, *Synechococcus *sp.; Toma, *Torpedo marmorata*.

To obtain information regarding the potential functional and structural similarities and differences of the predicted bacterial ART-LGIC and the animal receptors we prepared a multiple alignment of the bacterial sequences with the representatives of all the major classes of animal Cys-loop proteins (Figure 1) using the T_Coffee program [22]. The alignment was further refined on the basis of secondary structure predictions and comparisons with the available structure of the stand-alone animal LBD, ACHB. The multiple alignment shows that the majority of the highly conserved positions in the LBD are in the conserved strands, and when mapped onto the structure of ACHB, they correspond to the positions stabilizing the hydrophobic core of the β-sandwich (Figure 1, see also Additional data file 1). This observation strongly suggests that the bacterial versions would adopt a tertiary structure similar to the animal LBDs.

The bacterial LBDs differ notably from the animal LBDs, however, in lacking the characteristic cysteine residues which form the disulfide bridge in practically all known animal receptor subunits (Figure 1). However, in place of the second cysteine the bacterial sequences possess a highly conserved hydrophobic position that is likely to be buried in the hydrophobic core of the sandwich and, thereby, similarly stabilize the region corresponding to the Cys-loop of the animal sequences (Figure 1). This absence of the cysteines in the bacterial versions of these family is reminiscent of what was previously observed in the bacterial homologs of several animal extracellular protein domains, such as the SCP1/PR1 domain, the immunoglobulin domains and the MAC-perforin domains [23-25]. Eukaryotic cells typically possess an extensive secretory compartment, with a strongly oxidizing environment, in the form of the endoplasmic reticulum, through which a protein passes before secretion [26]. In contrast, in bacteria most disulfide bond formation occurs after extrusion to the periplasmic compartment [27]. The presence of this extensive secretory compartment in eukaryotic cells might have allowed a greater role for stabilization through disulfide bonds, and thereby favored the emergence of interacting cysteines in eukaryotic versions of domains as opposed to the bacterial counterparts.

Over and above the general conservation of hydrophobic residues in the 4-TM domain, there are some potential functionally relevant conserved positions shared by the bacterial and metazoan proteins. One of these is the helix-bending position in helix M1 (usually occupied by a proline (P), glycine (G) or serine (S), and corresponding to P221 in *Torpedo californica *ACHR α-chain), which is predicted to be critical for the flexibility of the structure to conformational change [13,28]. Another position of interest is in the middle of helix M2, and is occupied by a small residue (corresponding to S252 in *T. californica *ACHR α-chain) that initiates a bend in the helix resulting in the hydrophobic constriction or girdle that forms the channel gate [13]. Glycine 275 of *T. californica *ACHR α-chain, in the loop between helices M2 and M3, has been implicated as one the residues that may be critical for the rotational freedom of the ACHR M2 helix during the gating process [13]. The strong conservation of a small residue at this position in both the bacterial and animal members of this family suggests that it is likely to support this function throughout the superfamily. Less obvious is the role of a polar residue just before the start of helix M4 that is highly conserved across both bacterial and animal members of this superfamily. From its position in the structure, it is possible that interactions of residue with solvent water might play a role in stabilizing one of the conformational states.

One of the major determinants of ion selectivity is the sequence just amino-terminal to the helix M2 on the cytoplasmic side. The cation channels usually have a sequence motif of the form glutamate (E)-[arginine (R)/lysine (K)] with the glutamate playing a role in cation selection. The anion channels usually have a motif of the form alanine (A)-[RK] with the basic residue participating in anion selection [11,12,29]. A glutamate corresponding to that of the cation channels is seen in about eight of the bacterial sequences and a basic residue similar to the anion channels is seen in six of the bacterial sequences, suggesting that both selectivities are likely to be encountered in the bacterial sequences (Figure 1). In addition, like the animal sequences, the bacterial sequences contain poorly conserved polar or charged residues at the carboxyl terminus of the M2 helix, which might play a role in fine-tuning their selectivity [4,6,11,13]. The long hydrophilic linker between helices M3 and M4 is highly variable in length and sequence in the animal proteins. It has been implicated in cytoplasmic interactions with functional partners such as the P2X family of ATP receptors [30] and the cytoskeletal receptor-clustering protein gephyrin [31]. In contrast to the animal members of the superfamily, all bacterial versions possess an abbreviated cytoplasmic M3-M4 loop and are unlikely to have functional interactions that are seen in the animal versions.

**Figure 2:**
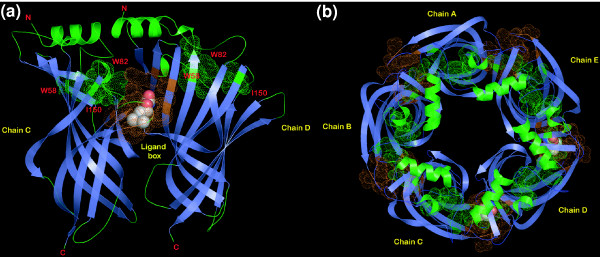
Cartoon representations of ACHB. **(a) **The ligand-binding dimer of ACHB; **(b) **the pentamer of ACHB. The representations were derived from the crystal structure of the snail acetylcholine-binding protein (PDB 1UV6). Residues forming the ligand-binding box are shaded orange. The chain of residues that could potentially act as a conduit for transmission of conformational changes is colored green and the prominent conserved ones among them have been labeled.

The ligand-binding box in ACHB has been termed the aromatic box as it is bounded by multiple aromatic residues (Figures 1, 2). In several metazoan receptors the positively charged group on the ligands has been suggested to form cation-π interaction with the π-orbitals of different aromatic residues in the binding box [32-34]. An examination of the ACHB structure [9] revealed that the side chains of eight residues almost completely envelop the ligand, and are the principal constituents of the ligand-binding box (Figure 2). Of these, the dyad of two consecutive cysteines, which are amino-terminal to the final strand of the LBD is observed only in a subset of the animal cation channels, and does not represent a conserved interaction position. Of the remaining six positions, two are from one of the subunits while the remaining four are from the other subunit (Table 1). The average number of aromatic residues in these positions in the bacterial proteins is 2.1, whereas in the animal sequences it is 2.6. Every sequence in our representative set, animal or bacterial, with the exception of the human Zn receptor [8], contains at least a single aromatic residue in one of these positions. This suggests that aromatic residues are critical for ligand interaction throughout this superfamily, though the exact position in the ligand-binding box that is occupied by an aromatic residue does not seem to be preserved. However, the smaller number of aromatic residues in the ligand-binding box of bacteria may indicate some differences in the type of ligand and the nature of the interactions.

Furthermore, an interesting difference is noted in the aromaticity of the positions corresponding to leucine (L) 112 (subunit D) and tryptophan (W) 143 (subunit C) of the ACHB structure between the bacterial and animal sequences (Figure 2). The ratio of aromatic residues at these positions is anti-correlated, and this anti-correlation is strongly preserved in the individual sequences. This suggests that these two positions might represent mutually exclusive, but functionally equivalent, surfaces for ligand interaction. The presence of at least one aromatic residue in most of the predicted ligand-binding pockets could imply that cation-π interactions with the bound ligand are widespread in the entire superfamily. However, other explanations are also possible. For example, one or more aromatic residues could have a possible structural role in constraining the pocket to favor a particular ligand or ligand orientation. Alternatively, they could provide the requisite hydrophobic environment in the pocket or interact with the ligand through aromatic stacking.

In addition to the residues discussed above, there are several other conserved residues in the LBD that may have a role in transmission of conformational changes. Among the most highly conserved features is the aPaD signature (where 'a' is any aromatic residue, and D is aspartate) in the middle of the region corresponding to the Cys-loop (Figure 1) and these residues are essential for wild-type receptor function [5]. They lie far away from the ligand-binding region and close to another nearly universally conserved basic residue at the end of the terminal strand of the LBD (Figure 1). This basic residue is known to be mutated in the glycine receptor α1 subunit in the human genetic disease sporadic hyperekplexia [35]. The aspartate from the aPaD motif and the basic residue could potentially form a salt bridge to stabilize the 'outer sheet' of the β sandwich and thereby regulate the preferential movement of the sheets after ligand binding. This proposal is consistent with recent studies that implicate some of these charged residues, especially the aspartate in the Cys-loop, in coupling ligand binding to further conformational changes leading to channel gating [36,37]. The other highly conserved positions are a tryptophan at the end of strand 2 (W58 in ACHB) and an aromatic or hydrophobic position (W82 in ACHB) that are in hydrophobic interaction with each other (Figures 1, 2). These residues are at the center of a set of fairly conserved positions (including D61, P84, D108, G109 and isoleucine (I) 150 in ACHB) in both bacterial and eukaryotic proteins that form a chain on either side from the ligand-binding box to the surface of the 'inner sheet' at the top of the LBD [9,28]. It is likely that these residues form a conduit for the propagation of the conformational change from the ligand-binding box to the inner sheet (Figure 2).

The conservation of certain key features in both the LBD and the 4-TM domains of the bacterial and eukaryotic receptors suggests that despite their extensive sequence divergence they are likely to share general functional and mechanistic properties. In the pentamer these residues appear to form a continuous ring passing through the top surface of the LBD, and undergo conformational changes in relation the presence of a bound ligand (Figure 2) [9].

### Functional significance of domain architectures and gene neighborhoods of bacterial ART-LGICs

**Figure 3:**
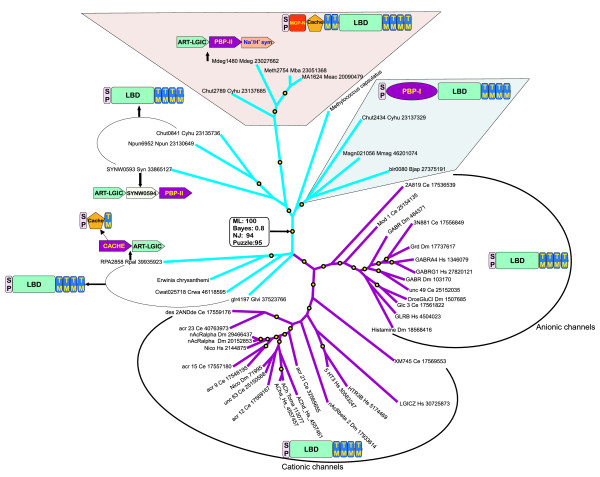
A phylogenetic tree of proteins containing the ART-LGIC domain with relevant domain architectures and gene neighborhoods. Bacterial branches are colored blue and animal branches magenta. Nodes with maximum-likelihood RELL bootstrap support of more than 70% are shown as yellow circles. Selected gene neighborhoods that provide contextual functional information are shown as box arrows. The globular domains in the domain architectures are drawn approximately to scale. LBD, the ligand-binding domain of the ART-LGIC domain. Species abbreviations are as in Figure 1.

We analyzed the domain architectures and gene neighborhoods of the predicted bacterial ART-LGICs to glean further insights regarding their biological functions. Unlike the animal ART-LGICs, the bacterial receptors show a greater diversity in their domain architectures, while preserving the core module which comprises the extracellular LBD and 4-TM channel-forming domain (Figure 3). The representatives from cyanobacteria, *Rhodopseudomonas *and one of the three versions from *C. hutchinsonii *show a simple architecture identical to the animal forms. Some versions, like those from the α-proteobacteria, *M. magnetotacticum *and *B. japonicum*, show a further amino-terminal fusion to a domain of the periplasmic binding protein type I (PBP-I) superfamily (Figure 3). The archetypal domains of the PBP-I superfamily are the bacterial proteins such as the lysine/arginine/ornithine-binding protein, that bind amino acids and other small molecules in the extracellular or periplasmic space and facilitate their uptake by ABC-family transporters [38]. Interestingly, PBP-I domains also form the LBDs of two distinct superfamilies of animal neuronal receptors. The NMDA-type receptors, which comprise a class of ligand-gated channels distinct from the ART-LGIC/Cys-loop superfamily, contain an amino-terminal PBP-I domain and a carboxy-terminal domain belonging to the second major superfamily of bacterial periplasmic binding proteins (the PBP-II superfamily, for example, HisJ) [39,40]. The channel-forming transmembrane domain in these proteins is inserted into the carboxy-terminal PBP-II domain. The metabotropic glutamate receptor and vertebrate taste receptors, which are G-protein-coupled receptors, contain a PBP-I domain amino-terminal to their 7-TM domains [39,40].

A third architectural theme in the bacterial ART-LGICs is a fusion of two additional amino-terminal domains to the core receptor module, namely the MCP-N (methyl-accepting chemotaxis protein-N domain) and Cache domains [41]. This version is seen in a number of phylogenetically distant prokaryotes, such as the archaeon *Methanosarcina *and the bacteria *Cytophaga *and *Microbulbifer *(Figure 3). The MCP-N and Cache domains are prevalent prokaryotic sensor domains that bind a variety of extracellular or periplasmic ligands and regulate signal transduction via a variety of carboxy-terminal signaling domains. In an interesting parallel to the PBP-I/II domains, the MCP-N and Cache domains are found in a regulatory subunit (α2-δ) of the animal voltage-gated calcium channels, and appear to comprise the binding site for the drug GABApentin and possibly an as-yet unknown endogenous ligand [41]. Thus, these architectures suggest that many of the predicted bacterial receptors might possess multiple ligand-interaction domains and that an interplay of allosteric effects could regulate their function. Remarkably, the additional domains found with the bacterial ART-LGIC proteins are also encountered in animal neuronal receptors, suggesting that all these domains belong to a common network of ancient sensory modules that have been utilized in diverse contexts [42].

Contextual information in the form of conserved gene neighborhoods or predicted operons in prokaryotes often provides hints to identify gene products that functionally or physically interact or belong to the same pathways or signaling cascades [43,44]. Accordingly, we examined the gene neighborhoods of all the predicted bacterial ART-LGICs to identify conserved neighborhoods or persistent patterns of genomic clustering of genes with similar functions. In some bacteria, the gene for the ART-LGIC was found in a conserved gene neighborhood along with a gene for a stand-alone version of the PBP-II superfamily (Figure 3). This is analogous to the above-noted fusion of the PBP-I domain to the ART-LGIC in other bacteria, and suggests that these stand-alone PBP-II domains probably functionally cooperate with the receptors. In one bacterium, namely *Rhodopseudomonas*, there is a similar predicted operon, but instead of a gene for a PBP-II superfamily protein, there is one for a stand-alone Cache domain. This situation parallels the fusion with the Cache domain in some of the receptor versions and these two independent proteins may similarly cooperate functionally.

Taken together, these observations suggest that bacterial ART-LGICs may function as chemotaxis receptors. As most bacterial genomes in which they are present contain only a single member of the ART-LGIC superfamily, it is likely that, in contrast to many of the well studied metazoan receptors, they function as homopentamers. The PBP-I, PBP-II MCP-N and Cache domains that are either fused or operonic with many of the predicted bacterial receptors may help in a preliminary concentration or sensing of amino acids or other small-molecule ligands. These ligands may then bind to the channel's LBD domain and activate an ionic flux across the cell membrane that in turn regulates the motility of the bacterium in response to the ligand. This proposal is analogous to the recently reported activity of a voltage-gated Na^+ ^channel in the bacterium *Bacillus pseudofirmus *in chemotaxis, motility and the regulation of the Na^+^-cycle [16]. Interestingly, in at least one bacterium, *Microbulbifer degradans*, the ART-LGIC with a predicted cation selectivity is in a predicted operon with a Na^+^/H^+ ^symporter, suggesting possible interactions with the Na^+ ^cycle.

### Phyletic patterns and phylogenetic relationships of the bacterial and eukaryotic ART-LGICs

Comparative genomics of ART-LGICs suggests that they show a highly non-uniform phyletic patterns. Among the eukaryotes they are only seen in animals, and could not be detected in the currently available genomic sequences of other crown-group eukaryotes such as plants, fungi, *Dictyostelium*, *Entamoeba*, apicomplexans or earlier-branching eukaryotic taxa such as *Giardia *and *Trichomonas*. Among the prokaryotes, too, they show a highly sporadic distribution: very distantly related taxa may possess similar receptors (for example, *Cytophaga *and the archaeon *Methanosarcina*, Figure 3), whereas closely related taxa may differ from each other in possessing or lacking a predicted ART-LGIC. These phyletic patterns are similar to those observed for several signaling receptors in prokaryotes and are suggestive of a high degree of mobility through lateral transfer, and frequent gene loss [45].

We constructed phylogenetic trees of the ART-LGICs by using an alignment that spanned the entire length of the LBD and the 4-TM channel domain, and included all bacterial members and representatives of all the major animal receptor groups. The trees constructed using several different methods - maximum likelihood, Bayesian inference, minimum evolution and neighbor-joining - produced congruent tree topologies (Figure 3). As expected, the tree showed a strongly supported monophyletic animal branch that in turn split up into the two major families corresponding to the great split between the classical acetylcholine-serotonin type (usually cationic) receptors and their relatives and the classical glycine-GABA type (usually anionic) receptors and their relatives [2,7,14,15].

All the animal sequences are much closer to each other to the exclusion of all other prokaryotic sequences (Figure 3). They possess several unique sequence and structure features, including the characteristic cysteines of the Cys-loop and the extra large variable region between the transmembrane helices M3 and M4. Its absence in the bacterial forms suggests that they are 'simpler' versions, which are closer to the primitive state. The mean intra-group distance of the metazoan versions, measured using the JTT substitution matrix on an alignment of 368 positions, is 1.7. This value is much lower than the intra-group distance of 3.01 that is observed for the bacterial forms (the overall mean distance being 2.8).

The prokaryotic proteins also show greater diversity of architectures in comparison to the stereotypic architecture of all the animal members of this superfamily. These observations suggest that the diversification of the prokaryotic forms preceded the emergence of the eukaryotic forms and thus that the root of the tree is more likely to lie in the bacterial lineage than within the metazoan lineage. Certain bacterial versions (those from *Crocosphaera*, *Gloeobacter*, *Erwinia *and *Rhodopseudomonas*) are markedly more similar in sequence to the eukaryotic forms (Figures 1, 3). Specifically, these similarities include the extension of strand 2 of the LBD, before the universally conserved W, and the hWxP motif (where h is a hydrophobic residue and x any residue) amino-terminal to strand 4 of the LBD. Constrained trees, where the animal branch was artificially grouped with the more distantly related bacterial sequences, were significantly worse (using the Kishino-Hasegawa and Bayesian posterior probability tests; data not shown) than the trees in which they were grouped with their closer bacterial homologs. This observation, taken together with the greater likelihood of the root being amongst the prokaryotes, suggests that the above features shared by some of the bacterial sequences and the animal versions are synapomorphies or derived characters.

Taken together, the phyletic patterns and the specific relationship of the animal sequences to a subset of the bacterial forms suggests that the common ancestor of the animal ART-LGICs probably arose via an early lateral gene transfer from a bacterium to the ancestral lineage leading to the modern metazoans. Following this transfer, the ancestral eukaryotic version acquired the characteristic cysteines of the Cys-loop and duplicated and diverged to give rise to the two major metazoan Cys-loop families. By the time of the common ancestor of the bilateral animals the two major families appear to have diversified into about nine distinct lineages (Figure 3). The biased sampling of eukaryotic genome sequences and the high frequencies of gene loss in the eukaryotes could imply that the transfer of the ART-LGICs from bacteria to the eukaryotes might have occurred well before the emergence of the animal lineage, and has been lost repeatedly in the other eukaryotes. While this possibility cannot be ruled out until more representative eukaryotic sequences become available, it is likely that there was a single precursor for all the animal sequences, which was acquired at some point from a bacterial source, and the massive radiation of the Cys-loop receptors occurred only after the animals branched off from the rest of the crown group. In principle it is possible that the bacterial sequences emerged through a secondary transfer from the animals. However, the potentially greater antiquity of the prokaryotic lineages possessing these proteins, combined with their greater diversity, makes this direction less likely given the current data. In addition, as discussed below, the case of the ART-LGIC receptors seems to fit the general pattern, which is observed for many other eukaryotic signaling proteins that appear to have a bacterial provenance.

It is of interest to note that several other animal neurotransmitter receptors show connections to bacterial signaling systems. In addition to the MCP-N and Cache domains shared by the metazoan voltage-gated Ca^2+ ^channels, and the PBP-I domains of various G-protein-linked and NMDA-type receptors, there are similar parallels in the receptors for the gaseous neurotransmitter nitric oxide (NO). The NO receptors of animals share two domains, namely the HNOB and HNOBA, which are involved in heme-dependent NO sensing with several bacterial signaling proteins [46]. Likewise, a recent analysis of the enzymes in the biosynthetic pathways of all common metazoan neurotransmitters suggested that many of them may have been laterally transferred from bacteria to eukaryotes at different points in eukaryotic evolution [47]. Some of these include some potentially late transfers, analogous to previous observations for the NO receptors and the present report on ART-LGICs. Furthermore, parallel instances of connections to bacterial sensory proteins have been noted in the case of plant receptors for cytokinin, ethylene and light (phytochromes), and certain small-molecule receptors of the cellular slime mold *Dictyostelium *(see [48] and references therein). Thus, the ART-LGICs appear to belong to a larger sensory network that probably first emerged in the bacterial signaling systems and was subsequently recruited by the eukaryotes in contexts unique to their own functional milieus.

## Conclusions

We report here the identification of several prokaryotic homologs of the animal acetylcholine receptor-type ligand gated ion channels (Cys-loop receptors). The pattern of the residues conserved in both the metazoan and bacterial receptors suggests that a common mechanism of channel-gating is likely to operate throughout this superfamily. Furthermore, the ligand-binding box appears to preserve at least one aromatic residue, although its exact position may not necessarily be conserved. The conservation pattern also suggests that a chain of positions leading out on either side from the ligand-binding box may mediate the transmission of the conformational change through the 'top' of the LBD, which may then transmit through the rest of the structure. The charge interactions between the acidic residue in the middle of the Cys-loop region and a basic residue the extreme carboxyl terminus of the LBD, just before the transmembrane domain also appear be universal features that might be involved in the process of channel gating. On the basis of the domain architectures and operon organizations, we predict that the bacterial ART-LGICs are likely to function as chemoreceptors for low-molecular-weight solutes in the environment. Phyletic and phylogenetic analyses suggest that the ancestor of the animal lineage probably acquired a single progenitor from a bacterial source, and it subsequently radiated to give rise to all the Cys-loop receptor subunits of the extant metazoans.

## Materials and methods

The nonredundant (NR) database of protein sequences (National Center for Biotechnology Information (NCBI)) was searched using the BLASTP program [49]. Unfinished microbial and eukaryotic genomes were searched using the TBLASTN program with protein queries [49]. Iterative database searches were conducted using the PSI-BLAST program with either a single sequence or an alignment used as the query, with a position-specific score matrix inclusion expectation (E) value threshold of 0.01 (unless specified otherwise); the searches were iterated until convergence [49]. For all searches with compositionally biased proteins, the statistical correction for this bias was used. Multiple alignments were constructed using the T_Coffee [22] or PCMA programs [22], followed by manual correction based on the PSI-BLAST results and structural information. All large-scale sequence-analysis procedures were carried out using the SEALS package [50]. Transmembrane regions were predicted in individual proteins using TMPRED [51], TMHMM2.0 [52] and TopPred II [53] with default parameters. For TopPred, the organism parameter was set to 'prokaryote' or 'eukaryote' depending on the source of the protein. Signal peptides were predicted using the SIGNALP program [54].

Protein structure manipulations were performed using the Swiss-PDB viewer program [55]. Protein secondary structure was predicted using a multiple alignment as the input for PHD [20] or JPRED2 [21]. Similarity-based clustering of proteins was carried out using BLASTCLUST [56].

Phylogenetic analysis was carried out using the maximum-likelihood, neighbor-joining, Bayesian inference and minimum evolution (least squares) methods. The MrBayes program was used for the Bayesian inference of phylogeny [57]. The alignment for phylogenetic analysis was prepared by visually deleting all those columns that contained non-conserved residues from five or fewer sequences. Regions with substantial gaps, which are replaced by numbers in Figure 1, were also entirely deleted from the alignment. The resulting alignment with 49 sequences and 368 columns was used for all subsequent phylogenetic analysis. Maximum-likelihood distance matrices were constructed with the TreePuzzle 5 program [58] using 1,000 replicates generated from the input alignment and used as the input for construction of neighbor-joining trees with the Weighbor program [59]. Weighbor uses a weighted neighbor-joining tree construction procedure that has been shown to correct effectively for long-branch effects.

The minimal evolution trees were constructed using the FITCH program [60] of the Phylip package on 1,000 bootstrap replicates prepared from the input sequence. For maximum-likelihood analysis two different procedures were used. In the first, a minimum evolution tree obtained using FITCH was provided as a input for the Protml program [61,62], which then produced a maximum-likelihood tree using local rearrangements. The statistical significance of the internal nodes of this maximum-likelihood tree was assessed using the relative estimate of logarithmic likelihood bootstrap (Protml RELL-BP) [61,62], with 10,000 replicates. In the second procedure an initial full maximum likelihood tree was constructed using the Proml program of the Phylip package [60]. A gamma distribution with one invariant and four variable sites with different rates was used for constructing this tree, which was then used as the guide tree to generate further full maximum-likelihood trees using the PhyML program with 100 bootstrap replicates generated from the input alignment [63]. The consensus of these 100 trees was derived using the Consense program of the Phylip package to obtain the bootstrapped full maximum-likelihood tree. Gene neighborhoods were determined by searching the NCBI PTT tables with a custom-written script. These tables can be accessed from the genomes division of the Entrez retrieval system.

## Additional data files

The following additional data are available with the online version of this paper. Additional data file 1 contains the conservation pattern of the ART-LGIC superfamily plotted onto the three-dimensional structure of the ACHB protein. Additional data file 2 contains the alignment of the proteins in Figure 1 in Word format.

## Supplementary Material

Additional data file 1 The conservation pattern of the ART-LGIC superfamily plotted onto the three-dimensional structure of the ACHB protein Click here for additional data file

Additional data file 2 The alignment of the proteins in Figure 1 Click here for additional data file

## Figures and Tables

**Table 1:** Character of residues in the conserved positions of the ligand-binding box

Ratio of aromatic residues							Overall aromaticity
Position*	R104(D)	L112(D)	W143(C)	T144(C)	Y185(C)	Y192(C)	
Bacteria	0.2	0.53	0.2	0.0	0.6	0.53	2.1
Animals	0.14	0.22	0.82	0.0	0.54	0.89	2.6

*The positions correspond to the D and C chains of ACHB (PDB: 1UV6)
