# Supplementary material for: Identification of the prokaryotic ligand-gated ion channels and their implications for the mechanisms and origins of animal Cys-loop ion channels
Source: Genome Biol. 2004 Dec 20;6(1):R4. doi: 10.1186/gb-2004-6-1-r4 (PMC549065; doi:10.1186/gb-2004-6-1-r4)
Supplement: Additional data file 1 — The conservation pattern of the ART-LGIC superfamily plotted onto the three-dimensional structure of the ACHB protein [file gb-2004-6-1-r4-s1.ppt]

## Slide 1
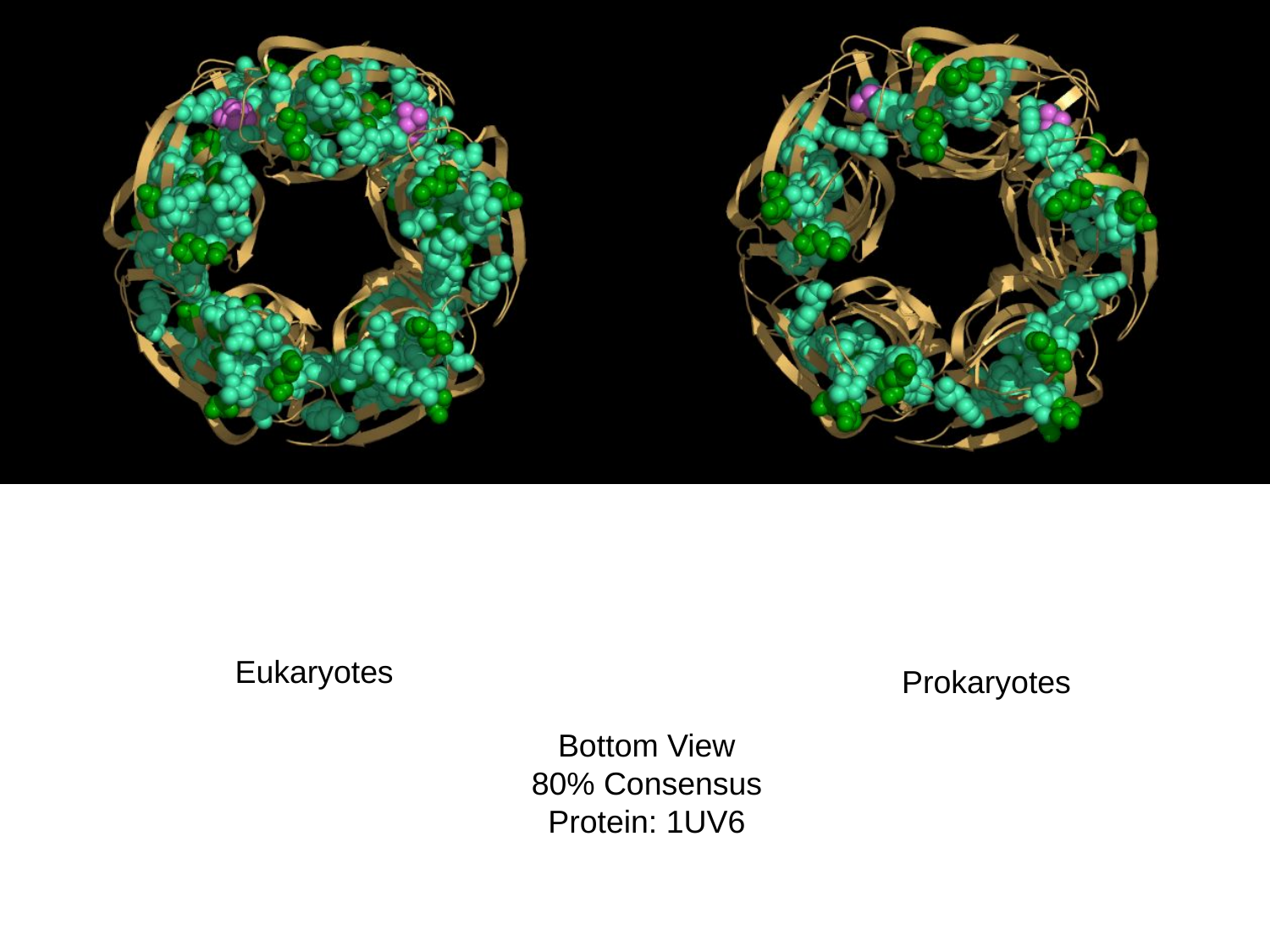

Eukaryotes
Prokaryotes
Bottom View
80% Consensus
Protein: 1UV6

## Slide 2
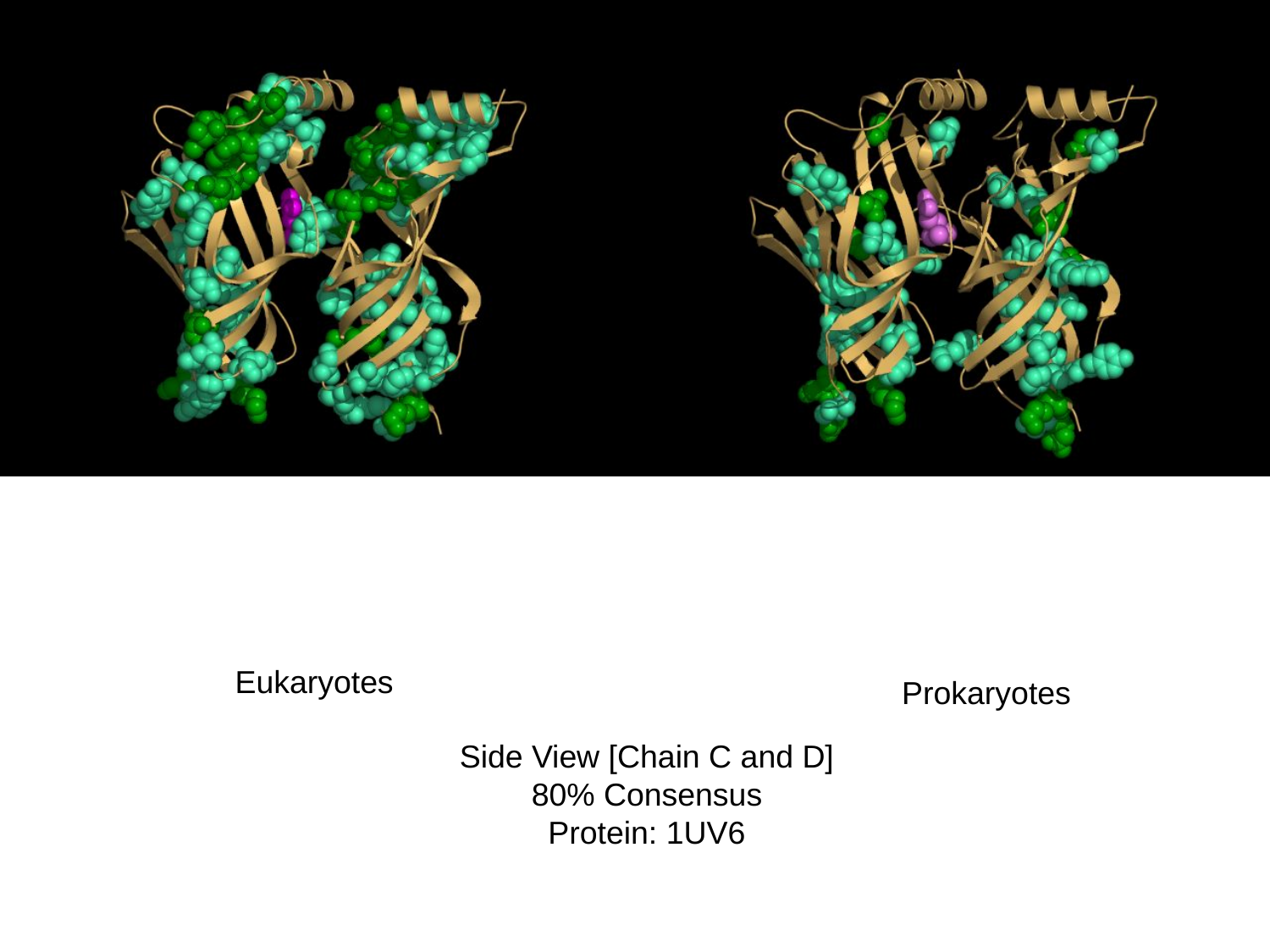

Eukaryotes
Prokaryotes
Side View [Chain C and D]
80% Consensus
Protein: 1UV6
