# Supplementary material for: Identification of the prokaryotic ligand-gated ion channels and their implications for the mechanisms and origins of animal Cys-loop ion channels
Source: Genome Biol. 2004 Dec 20;6(1):R4. doi: 10.1186/gb-2004-6-1-r4 (PMC549065; doi:10.1186/gb-2004-6-1-r4)
Supplement: Additional data file 2 — The alignment of the proteins in Figure 1 [file gb-2004-6-1-r4-s2.doc]

Secondary Structure --HHHHHHHHHH-----------------------EEEEEEEEEEEEEEEE---EEEEEEEEEEEEEEE-----------------EEEEEEE-------------EEEEEEEE---EEEE----------EEEEEE-EE-EEEEEEEEEEEEE-----------EEEEEEEEEE------EEEEE--------HH------EEEEE

Magn021056_Mmag_46201074 405 YVLK**EM**GERIAKG--QVTLVD**G**T-------PYHIVDV**I**SVG**V**D**V**I**RI**ND**VS**IKDMQ**W**DV**D**V**FM**---**W**F**K**WS-------------GGRLDVKDI---------E**K**I**S**AIN**A**V**K**-----ETSA---------IFK**E**DLT**HG**T-K**Y**RAYRKRLTLTAP**YDL**SN**FPFD**S**Q**T**L**P**LEI**AH**TN**-**K**NSTH**V**MLVPDT 5 VPVKDIKPQE**W**TYT

Mcap 1 LDRT**DF**KQQDQLIP-VDASDDQA------------IP**V**NLG**V**Y**V**E**NI**YN**FS**PNQKT**F**DA**E**G**WV**---**W**L**T**WPQ----------AAQDIFAVNGIPSS------Q**M**L**D**FVNSV**N**GWDFAMTPEYS--------EP**I**RLP**NG**S-Y**Y**QNFRYSHFYA**N**E**LNF**RQ**FPFQ**A**Q**T**F**E**LN**SED**EA**-**L**NAKH**V**RLIPDT 3 GTGEYIDIMG**Y**ITH

Npun6952_Npun_23130649 29 LTLII**I**VWLFVEVSEVSAETVLS-----------PQT**C**RTG**V**Y**V**A**SL**RD**LN**LAEKK**F**ST**D**F**YL**---**W**S**V**CPFKDLQPL----KSMKFLNVE-----------D**Y**KYFKA**A**Y**D**ST--LQRKDLP--------KW**F**YPK**EN**V-Y**W**SGRKIRATLY**Q**S**WNV**SN**FPFD**R**H**T**L**T**I**A**L**EE**TT**-**K**NSSQ**F**VYTPDF 3 GYQRNMDLDS**W**QIT

SYNW0593_Syn_33865127 1 AWRS**DA**PAAIDWG--TLMKAA**P**T-------APAEPLQQ-VG**A**Y**I**T**NI**SD**ID**LMDDQ**F**SI**E**L**LL**---**W**T**M**WHGD--------QDQNPSDQLR------------**V**L**N**GIYNG**D**I----QRFER---------IR**R**DQT**DG**H-S**W**SLYKVRSPVV**K**R**WRL**QR**YPFD**D**Q**L**L**H**VQI**GL**DD**-**P**LQPVNLDVVPK 3 SVTPSLLLPG**W**TLK

Chut0841_Cyhu_23135736 3 FINQ**KI**STALFFV--LLSLQL**S**A-------APEQPDT**V**RVGSY**I**L**SL**HD**IN**FHDKE**Y**TM**R**Y**WL**---**W**F**L**Y---------------DNPNFD------------**F**T**T**QVE**V**P**N**AK--SVEKPD---------VL**V**DTI**KG**K-T**W**VLMKMKSVMK**Q**S**WNV**ND**YPFD**E**Q**H**I**N**VSI**EN**TM**-**Y**DKRW**L**VYEIDS 3 TFAPTMNVDG**W**KIK

blr0080_Bjap_27375191 422 PDGI**DL**AAEQEKG--HVIAFE**D**R-------RYWIQRV**V**YTG**I**D**I**I**RV**SR**ID**VKQNS**F**NV**D**F**YL**---**W**M**R**FAG----------DDEAQTHVE------------**F**P**A**LLDRGAF-----DPARP--------IQ**A**GHE**DG**L-S**Y**RLYRINGDFKAH**FDL**HD**YPFD**T**Q**Q**L**H**L**L**F**QN**TE**-**Q**RREL**I**TYVIDR 13 EDGAYSGLPL**W**RFL

Chut2434_Cyhu_23137329 390 LFFT**QY**AGGRFH---SCPLQL**N**E-------YREVIPN**L**FFG**M**E**I**S**DI**YN**IN**MDENS**F**TS**D**F**YY**---**W**I**K**L------------DSNNRDAEK------------**Y**IIFQN**M**K**Q**N----ESSKE---------LI**F**EKT**DG**STI**Y**KLYKVSGIFYVN**YEL**EK**YPFD**A**Q**E**I**F**VRA**EI**LS**P**A**TKLK**V**SFDQKS 5 TKIDKFKITE**W**NKL

Chut2789_Cyhu_23137685 375 TVIS**DV**NGINTFI--NQTNGE**S**EI-----LHEDKPVY**I**PTG**I**Y**V**R**NI**EFK**D**G--RL**I**GL**N**GS**I**---**W**Q**K**L------------DTVLHKDVEPG--------VS**F**P**D**LSTDA**E**A----FNMEE---------VY**D**RIE**DG**H-R**V**IGWNFRLNIL**N**K**VDY**KL**YPFD**R**K**D**L**K**VNL**RH**HT**V**G**KNIF**L**VPDADA 10 GINKSIPLVG**W**DFL

Meth2754_Mba_23051368 377 IVVF**DI**ADVETVL--LNSDTN**P**-----------KAFR**I**PTG**V**F**I**Q**SI**EFS**T**S--ND**I**TM**T**G**YV**---**W**Q**N**I---------------SGLSVEK-----------**A**S**P**RFS**F**P**E**S----KESTV---------ER**D**YMD**ED**K-N**I**VGWRFTTILR**Q**Q**FDY**SR**YPFD**E**E**N**I**W**IKF**WN**NT**-**S**EESV**L**VPDFDS 10 GLENSLVLEG**W**KPQ

MA1624_Meac_20090479 375 FVVF**DM**AEVETVL--QHFSTD**S**-----------KTSR**I**PTG**V**F**L**E**TM**EFSGS--NE**I**IL**T**G**YV**---**W**Q**N**---------------FSGLDVDV-----------**A**S**P**GFS**F**P**E**S----KETTI---------ER**A**YVN**EN**E-S**V**VGWRFKTALR**Q**P**FDY**SR**YPFN**R**E**Y**V**W**IRF**WN**NA**-**S**EGNV**L**VPDFDS 10 GLEHSFVMEG**W**EPQ

Mdeg1480_Mdeg_23027662 633 VPVF**S**NEDALAYL--SIFNED**G**S--------EYSAQRHRLG**M**R**V**FVQSLDFNSANN**V**TM**T**G**YI**---**W**T**R**FP-----------DSFAGQDVSS-----------**L**T**P**VFPEA**E**S----VEFSNP--------IS**K**VDS**RG**N-I**H**VRWQFATTLR**Q**T**FDY**RK**YPFD**R**E**D**V**W**IRI**WP**ND**L**H**ENTV**L**MPEFSA 10 GVEEDIVLDG**W**QLV

Echr 1 GLPAWSAPADN-----AADAR**P**V-------------D**V**SVS**I**F**I**N**KI**YG**VN**TLEQT**Y**KV**D**G**YI**VAQ**W**TGKPRKTPGDKP---LIVENTQIERWI-----NNGL**W**V**P**ALE**F**I**N**VVGS-PDTGNK--------RL**M**LFP**DG**R-V**I**YNARFLGSFS**N**D**MDF**RL**FPFD**R**Q**Q**F**V**LEL**EP**FS**-**Y**NNQQ**L**RFSDIQ 3 ENIDNEEIDE**W**WIR

glr4197_Glvi_37523766 32 IGLLW**F**SPPVWGQ--DMVSPP**P**P-------IADEPLT**V**NTG**I**Y**L**I**EC**YS**LD**DKAET**F**KV**N**A**FL**SLS**W**K**D**RRLAFDP-------VRSGVRVKTYE-----PEAI**W**I**P**EIR**F**V**N**VENA-RDADVV--------DI**S**VSP**DG**T-V**Q**YLERFSARVL**S**P**LDF**RR**YPFD**S**Q**T**L**H**I**Y**L**IV**RS**-**V**DTRN**I**VLAVDL 3 GKNDDVFLTG**W**DIE

RPA2858_Rpal_39935923 12 FVAL**C**GTPASAAS--SPPEGL**P**E-------GVELPVK**V**RIG**L**R**V**L**DI**TE**IR**EVIGR**A**RLYV**EV**TQR**W**T**D**PRRRFDPLDA---GTSRIDRVGAEARQY--IAGI**W**T**P**GLA**I**D**N**QLGE-PRAKAD--------AV**S**VYS**DG**S-V**V**LVERYEADFRVG**VDM**AA**FPFD**R**Q**R**L**S**LSF**SL**PR**-**Y**AKQD**A**MLVTTE 6 RIEPKLSVID**W**RPL

Cwat025718_Crwa_46118595 17 KKKHW**F**IPHDQAI--PRPNDD**P**-----------EITQ**I**LVG**I**YTL**DL**AK**IN**EVEQT**V**YI**D**F**YL**GLQ**W**Y**D**SRFDSALSN------TNLSPYQRK------LEEV**W**Q**P**NLH**I**I**N**QRNL--DKELDE-------IV**H**INS**QG**I-V**T**YRQRYYGKLA**T**S**LDL**RR**FPFD**E**Q**T**I**K**IEL**IS**FS**-**Y**SPEE**I**HFVEAE 3 GISSEISLVN**W**SII

3N881_Ce_17556849 42 RYTT**KV**LDTILLN--QDKNFR**P**VN------PDNSPLQ**V**EVD**I**S**I**R**SM**GP**VS**EQNME**F**SL**D**C**YF**RQK**W**L**D**RRLAFTPIN----PSKPEIPLASKM-----LKDI**W**I**P**DTY**I**R**N**GRKSYLHTLTVPNI-----LF**R**VRS**DG**Q-V**H**VSQRLTIRSR**C**Q**M**F**L**KK**FPMD**T**Q**A**C**P**IEV**GS**LG**-**Y**FSKD**V**VYKWKD 3 DAKMGNTLSQ**Y**QVL

GABR_Dm_103170 57 VNISA**I**LDSFSVS--YDKRVR**P**N-------YGGPPVE**V**GVT**M**Y**V**L**SI**SS**VS**EVLMD**F**TL**D**F**YF**RQF**W**T**D**PRLAYRKR-----PGVETLSVGSEF-----IKNI**W**V**P**DTF**F**V**N**EKQSYFHIATTSNE-----FI**R**VHH**SG**S-I**T**RSIRLTITAS**C**P**MNL**QY**FPMD**R**Q**L**C**H**IEI**ES**FG**-**Y**TMRD**I**RYFWRD 5 GMSSEVELPQ**F**RVL

GABRA4_Hs_1346079 46 ENFT**RI**LDSLLDG--YDNRLR**P**G-------FGGPVTE**V**KTD**I**Y**V**T**SF**GP**VS**DVEME**Y**TM**D**V**FF**RQT**W**I**D**KRLKYDGP-------IEILRLNNMM-----VTKV**W**T**P**DTF**F**R**N**GKKSVSHNMTAPNK-----LF**R**IMR**NG**T-I**L**YTMRLTISAE**C**P**MRL**VD**FPMD**G**H**A**C**P**LKF**GS**YA**-**Y**PKSE**M**IYTWTK 7 VPKESSSLVQ**Y**DLI

Glc-3_Ce_17561822 25 SSDT**EI**IKKLLGKG-YDWRVR**P**PGINLTIPGTHGAVI**V**YVN**M**L**I**R**SI**SK**ID**DVNME**Y**SV**Q**LT**F**REE**W**V**D**GRLAYGFP------GDSTPDFLILTA----GQQI**W**M**P**DSF**F**Q**N**EKQAHKHDIDKPNV-----LI**R**IHR**DG**R-I**L**YSVRISMVLS**C**P**MHL**QY**YPMD**V**Q**T**C**L**IDL**AS**YA**-**Y**TEND**I**EYRWKK 6 KKGLHSSLPS**F**ELN

DrosGluCl_Dm_1507685 31 EKEK**KV**LDQILGAGKYDARIR**P**SGIN----GTDGPAI**V**RIN**L**F**V**R**SI**MT**IS**DIKME**Y**SV**Q**LT**F**REQ**W**T**D**ERLKFDDI-----QGRLKYLTTEL------ANRV**W**M**P**DLF**F**S**N**EKEGHFHNIIMPNV-----YI**R**IFP**NG**S-V**L**YSIRISLTLA**C**P**MNL**KL**YPLD**R**Q**I**C**S**LRM**AS**YG**-**W**TTND**L**VFLWKE 4 QVVKNLHLPR**F**TLE

unc-49_Ce_25152035 32 QLLS**SV**LDRLTNRTTYDKRLR**P**R-------YGEKPVD**V**GIT**I**H**V**S**SI**SA**VS**EVDMD**F**TL**D**F**YM**RQT**W**Q**D**PRLAFGSL-----DLGLSKEIDSLTVGVDYLDRL**W**K**P**DTF**F**P**N**EKKSFFHLATTHNS-----FL**R**IEG**DG**T-V**Y**TSQRLTVTAT**C**P**MDL**KL**FPMD**S**Q**H**C**K**LEI**ES**YG**-**Y**ETKD**I**DYYWGK 3 DLEITAVKFDTFQL

GABR_Dm_484371 38 ENVT**Q**TISNILQG--YDIRLR**P**N-------FGGEPLH**V**GMD**L**T**I**A**SF**DA**IS**EVNMD**Y**TI**T**M**YL**NQY**W**R**D**ERLAFNIFGQYFDDENDDGISDVLTLSGDFAEKI**W**V**P**DTF**F**A**N**DKNSFLHDVTERNK-----LV**R**LGG**DG**A-V**T**YGMRFTTTLA**C**M**MDL**HY**YPLD**S**Q**N**C**T**VEI**ES**YG**-**Y**TVSD**V**VMYWKP 3 RGVEDAELPQ**F**TII

GLRB_Hs_4504023 54 NSTS**NI**LNRLLVS--YDPRIR**P**N-------FKGIPVD**V**VVN**I**F**I**N**SF**GS**IQ**ETTMD**Y**RV**N**I**FL**RQK**W**N**D**PRLKLPSD-----FRGSDALTVDPT----MYKCL**W**K**P**DLF**F**A**N**EKSANFHDVTQENI-----LL**F**IFR**DG**D-V**L**VSMRLSITLS**C**P**LDL**TL**FPMD**T**Q**R**C**K**MQL**ES**FG**-**Y**TTDD**L**RFIWQS 3 VQLEKIALPQ**F**DIK

Histamine-_Dm_18568416 54 LSLP**DI**LPIPSKT--YDKNRA**P**K-------LLGQPTV**V**YLH**V**T**V**L**SL**DS**IN**EESMT**Y**VT**D**I**FL**AQS**W**R**D**PRPRLPEN------MSEQYRILDVD----WLHSI**W**R**A**DCF**F**K**N**AKKVTFHEMSIPNH-----YI**W**VYH**DK**T-L**F**YMSKLTLVLW**C**A**LKF**ES**YPHD**T**Q**I**C**S**M**M**I**ES**LS**-**H**TVED**L**VFIWNM 4 VVNTEIELPQ**L**DIS

2A819_Ce_17536539 28 GTEG**QI**VGRILSE--YDSSSR**P**PV------RDHADNS**A**ILV**I**TNIF**I**NR**L**IWHNNY**A**EV**D**L**YL**RQQ**W**Q**D**SRLKYDVD-----TREGIDEIRLPG-----NRKI**W**E**P**DTY**F**T**S**GKELSRNEKNSK-------HI**V**VEP**SG**Y-I**R**SSERVLLELPYA**Y**GTMF**PFTN**S**R**Q**F**T**IKL**GS**YN**-**Y**DIDD**I**VYLWAN 2 PLVNPIEVSQDLLK

GABRG1_Hs_27820121 62 GDIT**QI**LNSLLQG--YDNKLR**P**D-------IGVRPTV**I**ETD**V**Y**V**N**SI**GP**VD**PINME**Y**TI**D**I**IF**AQT**W**F**D**SRLKFNST-----MKVLMLNSNM-------VGKI**W**I**P**DTF**F**R**N**SRKSDAHWITTPNR-----LL**R**IWN**DG**R-V**L**YTLRLTINAE**C**Y**LQL**HN**FPMD**E**H**S**C**P**LEF**SS**YG**-**Y**PKNE**I**EYKWKK 5 ADPKYWRLYQ**F**AFV

Grd_Dm_17737617 107 ANIS**EL**LDNLLRG--YDNSIR**P**D-------FGGPPAT**I**EVD**I**M**V**R**SM**GP**IS**EVDMT**Y**SM**D**C**YF**RQS**W**V**D**KRLAFEGA-----QDTLALSVSM-------LARI**W**K**P**DTY**F**Y**N**GKQSYLHTITTPNK-----FV**R**IYQ**NG**R-V**L**YSSRLTIKAG**C**P**MNL**AD**FPMD**I**Q**K**C**P**LKF**GS**FG**-**Y**TTSD**V**IYRWNK 5 AIAEDMKLSQ**F**DLV

Mod-1_Ce_25154135 32 WSEG**KI**MNTIMSN--YTKML-**P**--------DAEDSVQ**V**NIE**I**H**V**Q**DM**GS**LN**EISSD**F**EI**D**I**LF**TQL**W**H**D**SALSFAHL------PACKRNITMETR---LLPKI**W**S**P**NTC**M**I**N**SKRTTVHASPSENV-----MV**I**LYE**NG**T-V**W**INHRLSVKSP**C**N**LDL**RQ**FPFD**T**Q**T**C**I**L**I**F**ES**YS**-**H**NSEE**V**ELHWME 3 TLMKPIQLPD**F**DMV

XM745_Ce_17569553 38 LAPK**RF**NYTVSL---YYLKLV---------EVIEPEK**V**SVV**L**E**M**A**EV**GR**LN**----VSTE**S**M**LV**FQY**W**Y**D**PRIAWDSS-----LYGDIKMLHMR------QDKV**W**S**P**TLS**L**F**R**INDIADFRDPDFR------MV**C**VEN**TG**H-T**Y**TTLSVKISLN**C**P**LDV**SM**FPYD**S**Q**T**C**R**IQF**NM**PL**-**F**FMQQ**V**EMFSQI 7 TVWEKMGNSE**W**ELA

HTR3B_Hs_5174469 30 SALY**HL**SKQLLQK--YHKEVR**P**VY------NWTKATT**V**YLD**L**F**V**HA**I**LD**VD**AENQI**L**KT**S**V**WY**QEV**W**N**D**EFLSWNSS-----MFDEIREISLP------LSAI**W**A**P**DII**I**N**E**FVDIERYPDLP--------YV**Y**VNS**SG**T-I**E**NYKPIQVVSA**C**S**LE**TYA**FPFD**V**Q**N**C**S**LTF**KS**IL**-**H**TVED**V**DLAFLR 7 DKKAFLNDSE**W**ELL

nAcRbeta-2_Dm_17933614 49 KALD**RL**HAGLFTN--YDSDVQ**P**--------VFQGTPTNVSLEM**V**V**TY**ID**ID**ELNGK**L**TT**H**C**WL**NLR**W**R**D**EERVWQPS-----QYDNITQITLK------SSEV**W**T**P**QIT**L**F**N**GDEGGL-MAET--------QV**T**LSH**DG**H-F**R**WMPPAVYTAY**C**E**LNM**LN**WPHD**K**Q**S**C**K**LKI**GS**WG**-**L**KVVL**P**ENGTAR 4 DHDDLVQSPE**W**EIV

5-HT3_Hs_30583247 38 PALL**RL**SDYLLTN--YRKGVR**P**VR------DWRKPTT**V**SID**V**I**V**YA**I**LN**VD**EKNQV**L**TTYI**WY**RQY**W**T**D**EFLQWNPE-----DFDNITKLSIP------TDSI**W**V**P**DIL**I**N**E**FVDVGKSPNIP--------YV**Y**IRH**QG**E-V**Q**NYKPLQVVTA**C**S**LDI**YN**FPFD**V**Q**N**C**S**LTF**TS**WL**-**H**TIQD**I**NISLWR 7 DRSVFMNQGE**W**ELL

LGICZ_Hs_30725873 31 AIWP**SL**FNVNLSKKVQESIQI**P**N-------NGSAPLL**V**DVR**V**F**V**S**NV**FN**VD**ILRYT**M**SSML**LL**RLS**W**L**D**TRLAWNTS------AHPRHAITLP------WESL**W**T**P**RLT**I**L**E**ALWVDWRDQSP--------QA**R**VDQ**DG**H-V**K**LNLALTTETN**C**N**FEL**LH**FPRD**H**S**N**C**S**LSF**YA**LS**-**N**TAME**L**EFQAHV VNEIVSVKRE**Y**VVY

nAChd_Hs_4557461 23 NEEE**RL**IRHLFQEKGYNKELR**P**VA------HKEESVD**V**ALA**L**T**L**S**NL**IS**LK**EVEET**L**TT**N**V**WI**EHG**W**T**D**NRLKWNAE-----EFGNISVLRLP------PDMV**W**L**P**EIV**L**E**N**NNDGSFQISYSC-------NV**L**VYHY**G**F-V**Y**WLPPAIFRSS**C**P**ISV**TY**FPFD**W**Q**N**C**S**LKF**SS**LK**-**Y**TAKE**I**TLSLKQ 15 DPEGFTENGE**W**EIV

acr-9_Ce_17548195 28 ADEY**RL**LADLRHN--YDPYER**P**VA------NASEPLV**V**SVK**I**Y**L**Q**QI**LD**VD**EKNQV**I**TLVA**WI**EYQ**W**T**D**YKLKWDPS-----EYGGIKDIRIP---GN-ANAI**W**K**P**DVL**L**Y**N**SADENFDSTYPV-------NY**V**VSY**TG**D-V**L**QVPPGILKLS**C**K**IDI**TY**FPFD**D**Q**I**C**H**LKF**GS**WT**-**Y**SGNF**I**DLRING 11 DVQYYVQNGE**W**NLL

acr-23_Ce_40763973 25 PIQY**EL**ANNIMEN--YQKGLI**P**KV------RKGSPIN**V**TLS**L**Q**L**Y**QI**IQ**VN**EPQQY**L**LL**N**A**WA**VER**W**V**D**QMLGWDPS-----EFDNETEIMAR------HDDI**W**L**P**DTT**L**Y**N**SLEMDDSASKKLTHVKL--TT**L**GKN**QG**AMV**E**LLYPTIYKIS**C**L**LNL**KY**FPFD**T**Q**T**C**R**MTF**GS**WS**-**F**DNSL**I**DYFPRT 6 GLANFLENDA**W**SVL

nAcRalpha-_Dm_45552371 311 YHEK**RL**LHDLLDP--YNTLER**P**VL------NESDPLQ**L**SFG**L**T**L**M**QI**ID**VD**EKNQL**L**VT**N**V**WL**KLE**W**N**D**MNLRWNTS-----DYGGVKDLRIP------PHRI**W**K**P**DVL**M**Y**N**SADEGFDGTYQT-------NV**V**VRN**NG**S-C**L**YVPPGIFKST**C**K**IDI**TW**FPFD**D**Q**R**C**E**MKF**GS**WT**-**Y**DGFQ**L**DLQLQD 4 DISSYVLNGE**W**ELL

nAcRalpha-_Dm_20152853 28 PHEK**RL**LNHLLST--YNTLER**P**VA------NESEPLE**V**KFG**L**T**L**Q**QI**ID**VD**EKNQI**L**TT**N**A**WL**NLE**W**N**D**YNLRWNET-----EYGGVKDLRIT------PNKL**W**K**P**DVL**M**Y**N**SADEGFDGTYHT-------NI**V**VKH**NG**S-C**L**YVPPGIFKST**C**K**MDI**TW**FPFD**D**Q**H**C**E**MKF**GS**WT**-**Y**DGNQ**L**DLVLNS 4 DLSDFITNGE**W**YLL

nAcRalpha-_Dm_45556050 59 PHEK**RL**LHALLDN--YNSLER**P**VV------NESDPLQ**L**SFG**L**T**L**M**QI**ID**VD**EKNQL**L**IT**N**I**WL**KLE**W**N**D**MNLRWNSS-----EFGGVRDLRIP------PHRL**W**K**P**DVL**M**Y**N**SADEGFDGTYAT-------NV**V**VRN**NG**S-C**L**YVPPGIFKST**C**K**IDI**TW**FPFD**D**Q**R**C**E**MKF**GS**WT**-**Y**DGFQ**L**DLQLQD 4 DISSFITNGE**W**DLL

Nico_Hs_2144875 24 EFQR**KL**YKELVKN--YNPLER**P**VA------NDSQPLT**V**YFS**L**S**L**L**QI**MD**VD**EKNQV**L**TT**N**I**WL**QMS**W**T**D**HYLQWNVS-----EYPGVKTVRFP------DGQI**W**K**P**DIL**L**Y**N**SADERFDATFHT-------NV**L**VNS**SG**H-C**Q**YLPPGIFKSS**C**Y**IDV**RW**FPFD**V**Q**H**C**K**LKF**GS**WS**-**Y**GGWS**L**DLQMQE 1 DISGYIPNGE**W**DLV

des-2ANDde_Ce_17559176 49 VPLV**RL**TRHLLSPERYDVRVR**P**IL------DHKKSLK**V**HIS**I**S**L**Y**QI**IE**VD**EPSQN**I**KL**N**V**WM**IQK**W**R**D**EYLDWNPN-----EYGMINSTIIP------FHHL**W**I**P**DTY**L**Y**N**SVKMSRDETERYMNIQATSNY**W**KGE**KG**AEL**S**FLYPAIYTIT**C**R**LNI**RF**FPYD**R**Q**N**C**T**LTI**SS**WT**-**N**SKSA**L**DYYADT 2 SMQSFIPNEE**W**QVK

unc-63_Ce_25150568 25 RDAN**RL**FEDLIAD--YNKLVR**P**VS------ENGETLV**V**TFK**L**K**L**S**QL**LD**VH**EKNQI**M**TT**N**V**WL**QHS**W**M**D**YKLRWDPV-----EYGGVEVLYVP------SDTI**W**L**P**DVV**L**Y**N**NADGNYQVTIMT-------KA**K**LTY**NG**T-V**E**WAPPAIYKSM**C**Q**IDV**EF**FPFD**R**Q**Q**C**E**MKF**GS**WT**-**Y**GGLE**V**DLQHRD 29 DLSDYYPSVE**W**DIL

Nico_Dm_71995 23 PDAK**RL**YDDLLSN--YNRLIR**P**VG------NNSDRLT**V**KMG**L**R**L**S**QL**ID**VN**LKNQI**M**TT**N**V**WV**EQE**W**N**D**YKLKWNPD-----DYGGVDTLHVP------SEHI**W**H**P**DIV**L**Y**N**NADGNYEVTIMT-------KA**I**LHH**TG**K-V**V**WKPPAIYKSF**C**E**IDV**EY**FPFD**E**Q**T**C**F**MKF**GS**WT**-**Y**DGYM**V**DLRHLK 12 DLQDYYISVE**W**DIM

nAcRalpha-_Dm_29466437 37 PHEK**RL**LHALLDN--YNSLER**P**VV------NESDPLQ**L**SFG**L**T**L**M**QI**ID**VD**EKNQL**L**IT**N**I**WL**KLE**W**N**D**MNLRWNSS-----EFGGVRDLRIP------PHRL**W**K**P**DVL**M**Y**N**SADEGFDGTYAT-------NV**V**VRN**NG**S-C**L**YVPPGIFKST**C**K**IDI**TW**FPFD**D**Q**R**C**E**MKF**GS**WT**-**Y**DGFQ**L**DLQLQD 4 DISSFITNGE**W**DLL

acr-21_Ce_32565655 80 QNVM**RL**YRDLLYD--YNNEVR**P**SV------HSKEPIN**V**TFV**F**S**L**T**QI**ID**VD**ERNQI**L**TT**N**S**WI**RLH**W**V**D**YKLVWDPR-----LYQNVTRIHIP------SDKI**W**K**P**DII**L**Y**N**NADAQYMKSVMST------DV**I**VDYL**G**N-I**H**WPLSAIFTSS**C**P**LDV**KH**YPFD**R**Q**T**C**I**LKY**AS**WA**-**Y**DGTK**I**DLLLKS 3 DLTNYITNTE**W**SLI

acr-15_Ce_17557180 20 PAEV**RL**INDLMSG--YVREER**P**TL------DSSKPVV**V**SLG**V**F**L**Q**QI**IN**LS**EKEEQ**L**EV**N**A**WL**KFQ**W**R**D**ENLRWEPT-----AYENVTDLRHP------PDAL**W**T**P**DIL**L**Y**N**SVDSEFDSSYKV-------NL**V**NYH**TG**N-I**N**WMPPGIFKVS**C**K**LDI**YW**FPFD**E**Q**V**C**Y**FKF**GS**WT**-**Y**TRDK**I**QLEKGD 1 DFSEFIPNGE**W**III

acr-12_Ce_17569167 36 DLES**QL**YEDLLFD--YNKVPR**P**VK------NSSDILT**V**DVG**A**S**L**I**RI**ID**VD**EKNQV**L**TT**N**L**WL**EMK**W**N**D**AKLTWTPE-----KYGGLKTLHIP------SDFI**W**T**P**DLV**L**Y**N**NAAGDPDITILT-------DA**L**VTF**EG**N-V**Y**WQPPAIYKSF**C**P**IDV**TW**FPYD**S**Q**K**C**E**MKF**GT**WT**-**Y**TGRY**V**DLKQLP 21 DLSFFYRSAE**W**DLL

nACha_Hs_4557457 22 EHET**RL**VAKLFKD--YSSVVR**P**VE------DHRQVVE**V**TVG**L**Q**L**I**QL**IN**VD**EVNQI**V**TT**N**V**RL**KQQ**W**V**D**YNLKWNPD-----DYGGVKKIHIP------SEKI**W**R**P**DLV**L**Y**N**NADGDFAIVKFT-------KV**L**LQY**TG**H-I**T**WTPPAIFKSY**C**E**I**I**V**TH**FPFD**E**Q**N**C**S**MKL**GT**WT**-**Y**DGSV**V**AINPES 3 DLSNFMESGE**W**VIK

ACh_Toma_113077 26 EHET**RL**VANLLEN--YNKVIR**P**VE------HHTHFVD**I**TVG**L**Q**L**I**QL**IN**VD**EVNQI**V**ET**N**V**RL**RQQ**W**I**D**VRLRWNPA-----DYGGIKKIRLP------SDDV**W**L**P**DLV**L**Y**N**NADGDFAIVHMT-------KL**L**LDY**TG**K-I**M**WTPPAIFKSY**C**E**I**I**V**TH**FPFD**Q**Q**N**C**T**MKL**GI**WT**-**Y**DGTK**V**SISPES 3 DLSTFMESGE**W**VMK

1UV6_Lst_47169299 1 LDRA**DI**LYNIRQT--SRPDVI**P**T-------QRDRPVA**V**SVS**L**K**F**I**NI**LE**VN**EITNE**V**DVVF**W**QQTT**W**S**D**RTLAWNSS-------HSPDQVSVP------ISSL**W**V**P**DLA**A**Y**N**AISKPEVLTPQ--------LA**R**VVS**DG**E-V**L**YMPSIRQRFS**C**D**VS**GVD**T-ES**GAT**C**R**IKI**GS**WT**-**H**HSRE**I**SVDPTT 4 DSEYFSQYSR**F**EIL

Consensus 90% .....h...............s...............h...h.l..h...p.....h..p.bh...W.p..................................h.s.....p.........................pG................hph..aPhD.p.h.h.h..........h....................a...

Key Residues ..................................................................*.*..................................*.............................@........@...............................##.*....*........................

**M1 Helix**

**M2 Helix**

**M3 Helix**

**M4 Helix**

Secondary Structure EEEEEEEEE----------------------EEEEEEEEEEEEE---HHHHHHHHHHHHHHHHHHHHHHH-------------HHHHHHHHHHHHHHHHHHHHHHHHHHHH------HHHHHHHHHHHHHHHHHHHHHHHHHH------------HHH---HHH-HH-HHHHHHHHHHHHHHHHHHHHHH--

Magn021056_Mmag_46201074 GRSVFS**D**LYR**Y**DSTFGDPDYRMGTGYKSPVY**F**ST**V**N**L**E**I**G**IKR**ILKPYL**F**TF**FLP**LL**I**ILG**I**ILII**L**W**V**P---LDQFA**PR**---I**NA**TISG**LIG**VLVYHMSQKNSFPKVGYT--MSA**D**Y**YF**LVA**Y**A**F**VVSMI**F**NIIFIQTLQSA GQKDVAKL**WN**----**-**-K**RL**SIGAMIAAIVI**Y**AAMTIFAMSVA 733\Bacterial

Mcap GSEIKTFIHN**Y**GTNFGLADSDG---EPTK-K**I**SQ**I**R**F**E**V**I**YKK**SITSSI**L**EL**FLP**LVTVMA**LV**MF**A**PM**L**S---SSLWD**VR**---L**GL**PPMV**LLT**LIFLQQGYKTELPDLPYV--TFL**D**T**IY**NLC**Y**LTTLILF**C**LFMWGSNKLDE 7 KVIAQINA**MD**----**-**-L**RF**QIGLTIALIGLGTINWFVVG--- 335|ART-LGIC

Npun6952_Npun_23130649 SFKIVE**Q**KVP**Y**ETTFGDPDLV----SPQD-S**Y**SR**L**V**I**S**I**G**IKR**VKFFSF**L**KLT**IG**VY**I**AFA**VA**ML**SF**F**Y**DSDQTSLAS**PR**---R**AI**YIGA**LFA**TLLNMRVQESVLGRTEDL--TLV**D**Q**I**HIAT**I**L**Y**VFGTG**V**VSVYSRLTSES GKKKQAIW**LD**R--R**V**FF**RL**FT**L**SFIVFNVI**A**IAHAIIVG--- 363|

SYNW0593_Syn_33865127 DPTGYA**S**SIS**L**MNDLGRPLADG---VAVR-R**Q**PT**V**S**F**D**L**P**IQR**RSLLFV**A**PD**FLG**YL**L**AIG**LC**CM**SL**L**I**T-------R**SR**---D**DL**ILAA**VVS**AGGNYVFIAGNLPVTAMT--GFI**G**N**L**QLII**F**LGILYVV**A**ADELIDNQLSL ----ISTR**FA**---K**G**L-**RV**LL**L**PSYVAMTL**L**GIWWIIP---- 300|

Chut0841_Cyhu_23135736 DFHVYRGQNE**Y**NTAFGDPRVT----STTS-E**Y**DT**F**N**I**K**M**T**LER**DAMGLF**M**KI**FLG**MY**I**AFF**IG**SI**SF**F**I**D--VK-EVE**SR**---F**AL**PVGG**LFA**AVGNKYIIDSLLPETSDY--TLV**D**T**L**HSIT**F**L**F**IFFTI**F**LNAYCVKLFEH NKAFRSQR**LN**Y--I**G**S-**RI**MM**L**SYILLNAF**F**VFMAAFY---- 321|

blr0080_Bjap_27375191 GLNYFV**E**SLS**S**GSTLGKAPLFGA--EART-E**F**AG**F**D**A**A**I**M**LRR**SSAIYM**L**KN**LLP**LF**L**LVL**VV**FA**TL**F**F**P---ETMFR**ER**---V**TI**PVTS**ILA**SAVLLVAVNSQIGDVGYT--VVVEE**MF**YIF**F**V**L**CLMAM**L**AGYRHEKLRDA GRKRVAVVS**D**---H**V**A-**QI**IY**A**GTVLAIIV**V**LYRRYAV---- 754|

Chut2434_Cyhu_23137329 KYYVTV**D**NEI**N**LGMYGDPDMEE---EKLY-E**F**KN**I**Y**F**R**L**N**VER**KQTTPL**L**EI**VLP**LV**L**IGL**IS**ISL**L**F**I**K----DISF**EN**L--GE**V**SIGV**FMS**IVAFSISFSASTPSADNL--TKA**D**Y**LF**WLT**F**I**V**VLLNF**M**IVILVNAIYEP ---EEVKN**ID**----**I**R-**KL**STGLGIGYIVL**V**SIVLLN----- 707|

Chut2789_Cyhu_23137685 GSYFSY**E**YKN**L**NTNFGLQHY-----QRQR-N**L**PE**L**S**F**N**I**I**LSR**KIIGAL**I**AH**ILP**LL**I**IQL**M**LFG**VI**V**I**F---SKTQV**EI**---S**GY**NTFG**VIN**SCAAFFFVIVISHIDLRNTLEIEMVT**Y**LEY**I**Y**F**IVYIY**V**LLVTVNALLFS -SAKHYAF**VD**YNNN**Y**IP**KL**IF**W**PLFMLASI**L**ITLVLFY---- 709|

Meth2754_Mba_23051368 KTFFSY**R**MNS**Y**NTNFGVKDF------KHR-N**L**SE**L**Y**F**N**V**A**IKR**DLKSPF**V**SD**LLP**II**V**VAI**L**LFV**VL**L**I**T----TREE**EK**NQ-F**GF**KSSG**VLT**YCASLFFVLIVSHASLRAKIPTN**C**M**IY**LEY**F**Y**L**ILYMA**I**LGVSLNSIVFA -SHMNIPF**ID**TKDN**L**YV**KV**LY**W**PIITGFLL**I**ITLLNFY---- 698|

MA1624_Meac_20090479 KTFFSY**R**VNS**Y**NTDFGVGDF------THS-N**V**PE**L**Y**F**N**I**E**IKG**NFKDPF**V**SN**LLS**VI**V**ISI**L**LFA**VL**T**I**T-----TRD**EK**KTLF**SF**SSSG**VLS**YCSSLFFVLIVAHASLRTRTAMH**G**I**IY**LEY**F**Y**F**IMYMA**I**LAVSLNSIVFG -SNMDIRF**IN**AKDN**L**YV**KL**LY**W**PVILGCLL**L**ITLLNFY---- 696|

Mdeg1480_Mdeg_23027662 SSHFSY**K**KNN**Y**NTALDTVG------SGNN-A**I**PE**L**Y**F**N**V**G**L**A**R**LFVDPF**I**AD**MLP**IV**V**VCL**LV**FA**VL**L**I**TT-VKAGDI**EL**---K**GF**SSAN**VLS**YCAALYFVLIVSHVHLRETLNAF**G**I**IY**LEI**F**Y**F**CMYFI**I**LIVSANSLAIT -SEKTPAF**I**RNNDN**Y**IA**RL**CY**F**PFITLTLL**I**ATIWMFY---- 966|

Echr 1KASTHI**S**DIR**Y**DHLSSVQ-------PNQN-E**F**SR**I**T**V**R**I**D**A**V**R**NPSYYL**W**SF**ILP**LG**L**IIA**AS**WS**VF**W**L**E-----SFS**ER**---LQTSFTL**MLT**VVAYAFYTSNILPRLPYT--TVI**D**Q**MI**IAG**Y**GSIFAAI**L**LIIFAHHRQAN -GVEDDLL**I**Q---R**C**--**RL**-A**F**PLGFLAIG**C**VLVIRGITL-- 328|

glr4197_Glvi_37523766 SFTAVV**K**PAN**F**ALE-------------DR-L**E**SK**L**D**Y**Q**L**R**ISR**QYFSYIPNI**ILP**ML**F**ILF**IS**WT**AF**WST-----SY-**EA**N--V**TL**VVST**LIA**HIAFNILVETNLPKTPYM--TYT**G**A**II**FMI**Y**L**F**YFVAV**I**EVTVQHYLKVE SQPARAAS**IT**---R**A**S-**RI**-A**F**PVVFLLAN**I**ILAFLFFGF-- 359|

RPA2858_Rpal_39935923 DLTFFY**D**EAA**G**WN--------------AR-A**Y**SR**L**N**A**T**I**G**IER**LSERYL**L**RL**FIP**IVSTLA**VS**LF**VL**W**I**P-----GTA**PK**D--H**G**SLVFS**AL**LALAAISFTYEASFPGSISLNTPI**A**K**II**SLG**Y**F**Y**LVVVV**L**IDALLWKPRSD 1 ASRYHVLA**IG**L--R**S**HC**RW**-A**L**PSIMVIVC**L**ALVLRGLPG-- 354|

Cwat025718_Crwa_46118595 GLSSHS**H**AHY**L**QPE-------------QQ-D**Y**AR**F**D**Y**E**I**K**VKR**HSSFYA**W**RV**LFP**VA**L**IVF**MS**DL**VF**W**L**E---PTQII**PQ**---I**TL**ATAT**MVS**LITYQFILRQELPKMNYL--TAE**D**K**VI**VGS**M**L**L**VFIAL**V**KSVTSINLVAG GYRELALS**LD**---D**I**L-**K**NPDSALQKVALNTTSLGLIIKYNC 321/

3N881_Ce_17556849 SLSKSE**R**NVS**D**F-------------RFSDRN**I**SV**L**N**V**Y**F**K**LQR**QQGYYI**L**QI**Y**T**P**CT**L**VVV**MS**WV**SF**W**I**N---KEASP**AR**---V**SL**GIMT**VLS**MSTIGFGLRTDLPKVSHS--TAL**D**V**YI**LTC**F**V**F**LFAAM**V**EYAVINYAQIV 103 DPAEVVNK**ID**---N**F**S-**KL**-A**F**PTLYIIFN**V**FYWVAYLHLIP 485\Eukaryotic

GABR_Dm_103170 GHRQRA**T**EIN**L**T---------------TG-N**Y**SR**L**A**C**E**I**Q**F**V**R**SMGYYL**I**QI**YIP**SG**L**IVV**IS**WV**SF**L**A**Q---SQCNA**GA**---C**AL**GVTT**VLT**MTTLMSSTNAALPKISYV--KSI**D**V**YL**GTC**F**V**M**VFASL**L**EYATVGYMAKR 200 LLGITPSD**ID**---K**Y**S-**RI**-V**F**PVCFVCFN**L**MYWIIYLHVSD 594|Anionic

GABRA4_Hs_1346079 GQTVSS**E**TIK**S**I---------------TG-E**Y**IV**M**T**V**Y**F**H**LRR**KMGYFM**I**QT**YIP**CI**M**TVI**LS**QV**SF**W**I**N---KESVP**AR**---T**VF**GITT**VLT**MTTLSISARHSLPKVSYL--TAM**D**W**FI**AVC**F**A**F**VFSAL**I**EFAAVNYFTNI 163 PSGSGTSK**ID**---K**Y**A-**RI**-L**F**PVTFGAFN**M**VYWVVYLSKDT 546|ART-LGIC

Glc-3_Ce_17561822 NVDTTL**C**TSK**T**N---------------TG-T**Y**SC**L**RTV**L**E**LRR**QFSYYL**L**QL**YIP**ST**M**LVI**VS**WV**SF**W**L**D---RGAVP**AR**---V**TL**GVTT**LLT**MTTQASGINAKLPPVSYT--KAI**D**V**WI**GAC**L**T**F**IFGAL**L**EFAWVTYISSR 109 NVDDNAKR**AD**---L**I**S-**RV**-L**F**PTLFVCFN**F**VYWTKYSQYHA 480|

DrosGluCl_Dm_1507685 KFLTDY**C**NSK**T**N---------------TG-E**Y**SC**L**K**V**D**L**L**FRR**EFSYYL**I**QI**YIP**CC**M**LVI**VS**WV**SF**W**L**D---QGAVP**AR**---V**SL**GVTT**LLT**MATQTSGINASLPPVSYT--KAI**D**V**W**TGVC**L**T**F**VFGAL**L**EFALVNYASRS 82 RQCSRSKR**ID**---V**I**S-**RI**-T**F**PLVFALFN**L**VYWSTYLFREE 453|

unc-49_Ce_25152035 PQFQPTLYFV**N**T---------TKAETSSG-K**Y**VR**L**A**L**E**V**I**L**V**R**NMGFYT**M**NI**VIP**SI**L**IVT**IS**WV**SF**W**L**N---REASP**AR**---V**GL**GVTT**VLT**MTTLITTTNNSMPKVSYV--KGL**D**V**FL**NFC**F**V**M**VFASL**L**EYAIVSYMNKR 110 CQRWTPAK**ID**---K**L**S-**RY**-G**F**PLSFSIFN**I**VYWLYMKYLSL 490|

GABR_Dm_484371 GYETND**R**KER**L**A---------------TG-V**Y**QR**L**S**L**S**F**K**LQR**NIGYFV**F**QT**YLP**SI**L**IVM**LS**WV**SF**W**I**N---HEATS**AR**---V**AL**GITT**VLT**MTTISTGVRSSLPRISYV--KAI**D**I**YL**VMC**F**V**F**VFAAL**L**EYAAVNYTYWG 115 PKIKDVNI**ID**---K**Y**S-**RM**-I**F**PISFLAFN**L**GYWLFYILE-- 496|

GLRB_Hs_4504023 KEDIEYGNCT**K**Y------------YKGTG-Y**Y**TC**V**E**V**I**F**T**LRR**QVGFYM**M**GV**YAP**TL**L**IVV**LS**WL**SF**W**I**N---PDASA**AR**---V**PL**GIFS**VLS**LASECTTLAAELPKVSYV--KAL**D**V**WL**IAC**L**L**F**GFASL**V**EYAVVQVMLNN 108 VIPTAAKR**ID**---L**Y**A-**RA**-L**F**PFCFLFFN**V**IYWSIYL---- 497|

Histamine-_Dm_18568416 NNYTTD**C**TIE**Y**S---------------TG-N**F**TC**L**A**I**V**F**N**LRR**RLGYHL**F**HT**YIP**SA**L**IVV**MS**WI**SF**W**I**K---PEAIP**AR**---V**TV**GVTS**LLT**LATQNTQSQQSLPPVSYV--KAI**D**I**WM**SSCSV**F**VFLSL**M**EFAVVNNFMGP 40 HGHATAIY**ID**---K**F**S-**RF**-F**F**PFSFFILN**I**VYWTTFL---- 426|

2A819_Ce_17536539 GDLTFE**E**ASA**G**D---------CVGNYTVG-V**Y**SC**I**D**A**H**V**Y**FSA**STISGL**M**SW**FLP**SL**F**LLIG**S**WLH**F**W**I**H--------**GS**---W**SV**PRTIS**AA**VPFFILAAYYIFMREDSY-TQAQ**G**A**WL**AFC**L**V**L**TFFSF**V**EYFLVICCGGR 31 ASFRDNNG**ID**---V**I**S-**RV**-A**F**PIVTIVFL**I**IYFIFIV---- 390|

GABRG1_Hs_27820121 GLRNST**E**ITH**T**I---------------SG-D**Y**VI**M**T**I**F**F**D**LSR**RMGYFT**I**QT**YIP**CI**L**TVV**LS**WV**SF**W**I**N---KDAVP**AR**---T**SL**GITT**VLT**MTTLSTIARKSLPKVSYV--TAM**D**L**FV**SVC**F**I**F**VFAAL**M**EYGTLHYFTSN 70 RIHIRIAK**ID**---S**Y**S-**RI**-F**F**PTAFALFN**L**VYWVGYLYL-- 465|

Grd_Dm_17737617 72GSTTGL**S**GTI**T**L-----------ETNHPS-E**Y**SM**L**M**V**N**F**H**LQR**HMGNFL**I**QV**Y**G**P**CC**L**LVV**LS**WV**SF**W**L**N---REATA**DR**---V**SL**GITT**VLT**MTFLGLEARTDLPKVSYP--TAL**D**F**FV**FLS**F**G**F**IFATI**L**QFAVVHYYTKY 157 PQYNSVSK**ID**---R**A**S-**RI**-V**F**PLLFILIN**V**FYWYGYLSRSS 675|

Mod-1_Ce_25154135 HYSTKK**E**TLL**Y**P---------------NG-Y**W**DQ**L**Q**V**T**F**T**FKR**RYGFYI**I**QA**YVP**TY**L**TII**VS**WV**SF**C**M**E---PKALP**AR**---T**TV**GISS**LLA**LTFQFGNILKNLPRVSYV--KAM**D**V**WM**LGC**I**S**F**VFGTM**V**ELAFVCYISRC 118 LARFHPEA**VD**---K**F**S-**IV**-A**F**PLAFTMFN**L**VYWWHYLSQTF 484/

XM745_Ce_17569553 NLTHSV**E**LLS**Y**G-------------DGLG-D**M**QL**A**T**F**E**I**R**IRR**NPMYYI**Y**MI**IFP**SF**I**INA**LS**II**GV**F**L**K---KTDKM**SK**---L**NV**GLTN**IMT**MTFILGVMADKIPKTGSI--PLL**G**I**YI**IVN**L**F**I**MIVAVGLTIVLAEIQKC 15 LEYVLGEP**L**E---T**I**C-**MV**-I**L**EIFNTAIF**M**VMIGFWINDI- 385\Eukaryotic

HTR3B_Hs_5174469 SVSSTY**S**ILQ**S**S---------------AG-G**F**AQ**I**Q**F**N**V**V**MRR**HPLVYV**V**SL**LIP**SI**F**LML**VD**LG**SF**Y**L**P----PNCR**AR**---I**VF**KTSV**LVG**YTVFRVNMSNQVPRSVGS-TPLI**G**H**FF**TIC**M**A**F**LVLSL**A**KSIVLVKFLHD 74 EWLVLLSR**FD**---R**L**L-**F**Q-S**Y**LFMLGIYT**I**TLCSLWALWGG 440|Cationic

nAcRbeta-2_Dm_17933614 ----DS**R**AHF**V**S---------------QD-Y**Y**GY**M**E**Y**T**L**T**AQR**RSSMYT**A**VI**Y**T**P**AS**C**IVI**LA**LS**AF**W**L**P---PHMGG**EK**---IM**I**NGLL**IIV**IAAFLMYFAQLLPVLSNN-TPLV**V**I**FY**STS**L**L**Y**LSVST**I**VEVLVLYLATG 72 DWALLATA**VD**---R**I**S-**FV**-S**F**SLAFLILA**I**RCSV------- 441|ART-LGIC

5-HT3_Hs_30583247 GVLPYF**R**EFS**M**E--------------SSN-Y**Y**AE**M**K**F**Y**V**V**IRR**RPLFYV**V**SL**LLP**SI**F**LMV**MD**IV**GF**Y**L**P----PNSG**ER**---V**SF**KITL**LLG**YSVFLIIVSDTLPATAIG-TPLI**G**V**YF**VVC**M**A**L**LVISL**A**ETIFIVRLVHK 108 DWLRVGSV**LD**---K**L**L-**F**H-I**Y**LLAVLAYS**I**TLVMLWSIWQY 483|

LGICZ_Hs_30725873 DLKTQVPPQQ**L**--------------------**V**PC**F**Q**V**T**L**R**LKN**TALKSI**I**AL**LVP**AE**A**LLL**AD**VC**G**GL**L**P----LRAI**ER**---I**GY**KVTL**LLS**YLVLHSSLVQALPSSSSC-NPLLIY**YF**TIL**L**L**L**LFLST**I**ETVLLAGLLAR 37 SQRSWPET**AD**---R**I**F-**FL**-V**Y**VVGVLCTQ**F**VFAGIWMWAAC 393|

CHRND_Hs_4557461 HRPARV**N**VDP**R**A------------PLDSP-S**R**QD**I**T**F**Y**L**I**IRR**KPLFYI**I**NI**LVP**CV**L**ISF**MV**NL**VF**Y**L**P----ADSG**EK**---T**SV**AISV**LLA**QSVFLLLISKRLPATSMA-IPLI**G**K**FL**LFG**M**V**L**VTMVV**V**ICVIVLNIHFR 124 SWNRVART**VD**---R**L**C-**LF**-V**V**TPVMVVGT**A**WIFLQGVYNQP 496|

acr-9_Ce_17548195 AVPARH**E**TNI**F**D---------------EQ-P**Y**PS**L**F**F**Y**L**I**IQR**RTLYYG**L**NL**IIP**SF**L**ISL**MT**VL**GF**T**L**P----PDAG**EK**---I**TL**EITI**LLS**VCFFLSMVADMTPPTSEA-VPLI**G**L**II**FSG**A**F**F**SCCML**V**VSASVVFTVLV 171 DWKFAAMV**VD**---R**C**C-**LI**-T**F**SVFIVVST**C**GIMFSSPHLIA 542|

acr-23_Ce_40763973 GTKVNR**E**EKK**Y**T------------**CC**-PV-N**Y**TL**L**H**Y**D**V**V**IQR**KPLYYV**L**NL**IAP**TA**V**ITF**IS**II**GF**FTSVNVHDLRQ**EK**---I**TL**GITT**LLS**MSIMIFMVSDKMPSTSTC-VPLI**A**L**FY**TLM**I**T**I**ISVGT**L**AASSVIFVQKL 154 EWDWVAAV**L**E---R**V**F-**LI**-F**F**TICFLFSA**I**GINLYGWYIWY 538|

nAcRalpha-_Dm_45552371 GVPGKR**N**EIY**Y**N------------**CC**-PE-P**Y**ID**I**T**F**A**I**I**IRR**RTLYYF**F**NL**IIP**CV**L**IAS**MA**LL**GF**T**L**P----PDSG**EK**---L**SL**GVTI**LLS**LTVFLNMVAETMPATSDA-VPLL**G**T**YF**NCI**M**F**M**VASSV**V**STILILNYHHR 160 DWKFAAMV**VD**---R**L**C-**LI**-I**F**TMFTILAT**I**AVLLSAPHIIV 807|

nAcRalpha-_Dm_20152853 AMPGKK**N**TIV**Y**A------------**CC**-PE-P**Y**VD**I**T**F**T**I**Q**IRR**RTLYYF**F**NL**IVP**CV**L**ISS**MA**LL**GF**T**L**P----PDSG**EK**---L**TL**GVTI**LLS**LTVFLNLVAESMPTTSDA-VPLI**G**T**YF**NCI**M**F**M**VASSV**V**LTVVVLNYHHR 129 DWKFAAMV**VD**---R**F**C-**LI**-V**F**TLFTIIAT**V**TVLLSAPHIIV 522|

nAcRalpha-_Dm_45556050 GVPGKR**N**EIY**Y**N------------**CC**-PE-P**Y**ID**I**T**F**A**I**L**IRR**KTLYYF**F**NL**IVP**CV**L**IAS**MA**LL**GF**T**L**P----PDSG**EK**---L**SL**GVTI**LLS**LTVFLNMVAETMPATSDA-VPLL**G**T**YF**NCI**M**F**M**VASSV**V**STILILNYHHR 164 DWKFAAMV**VD**---R**L**C-**LI**-I**F**TLFTIIAT**L**AVLFSAPHFIF 559|

Nico_Hs_2144875 GIPGKR**S**ERF**Y**E------------**CC**-KE-P**Y**PD**V**T**F**T**V**T**MRR**RTLYYG**L**NL**LIP**CV**L**ISA**LA**LL**VF**L**L**P----ADSG**EK**---I**SL**GITV**LLS**LTVFMLLVAEIMPATSDS-VPLI**A**Q**YF**AST**M**I**I**VGLSV**V**VTVIVLQYHHH 138 EWKFAACV**VD**---R**L**C-**LM**-A**F**SVFTIICT**I**GILMSAPNFVE 495|

des-2ANDde_Ce_17559176 SFKIHR**H**EYK**Y**A------------**CC**-AE-P**W**VI**L**Q**A**S**L**V**IQR**KPLYYL**V**NL**IIP**TS**I**ITL**VA**IT**GF**FTPASTDDDRT**EK**---I**NL**GITT**LLA**MSILMLMVSDQMPTTSEF-VPLI**A**W**FY**LSI**I**I**I**ISIGT**F**LTSVVLSVQGR 155 EWEFLATV**LD**---R**F**L-**LI**-V**F**VGAVVIVT**A**GLILVGRMAQY 552|

unc-63_Ce_25150568 NVPGKR**H**SKR**Y**P------------**CC**-ES-P**F**ID**I**T**Y**E**I**H**LRR**KTLFYT**V**NL**IFP**SVGISF**LT**AL**VF**Y**L**P----SDGG**EK**---I**SL**CISI**LIS**LTVFFLLLVEIIPSTSLV-IPLI**G**K**YL**LFT**M**V**L**VTLSV**V**VTVVTLNVHYR 110 DWKYISVV**MD**---R**I**F-**LI**-T**F**TFACAFGT**V**VIIARAPSIYD 496|

Nico_Dm_71995 RVPAVR**N**EKF**Y**S------------**CC**-EE-P**Y**LD**I**V**F**N**L**T**LRR**KTLFYT**V**NL**IIP**CVGISF**LS**VL**VF**Y**L**P----SDSG**EK**---I**SL**CISI**LLS**LTVFFLLLAEIIPPTSLT-VPLL**G**K**YL**LFT**M**M**L**VTLSV**V**VTIAVLNVNFR 172 DWKYVAMV**LD**---R**M**F-**LW**-I**F**AIACVVGT**A**LIILQAPSLHD 539|

nAcRalpha-_Dm_29466437 GVPGKR**N**EIY**Y**N------------**CC**-PE-P**Y**ID**I**T**F**A**I**L**IRR**KTLYYF**F**NL**IVP**CV**L**IAS**MA**LL**GF**T**L**P----PDSG**EK**---L**SL**GVTI**LLS**LTVFLNMVAETMPATSDA-VPLL**G**K**YF**NCI**M**F**M**VASSV**V**STILVLNYHHR 164 DWKFAAMV**VD**---R**L**C-**LI**-I**F**TLFTIIAT**L**AVLFSAPHFIV 537|

acr-21_Ce_32565655 GIRAEK**N**QVI**Y**S------------**CC**-PE-P**Y**PF**I**D**V**H**V**T**IER**RAMFYV**F**NL**ILP**CV**L**ISL**IA**LM**GF**Y**M**P----TDSG**EK**---V**TL**GITS**LLS**TTVFLMMVAEGMPPTAEA-LPLI**G**I**YF**GVT**I**M**L**VALGT**A**MTVFTVNIHHT 130 AKKKVGSI**VT**T-LN**Y**C-**LY**II**L**TFKITGSRSCIEYCPKYKNN 549|

acr-15_Ce_17557180 DYRTNI**T**VKQ**Y**E------------**CC**-PE-Q**Y**ED**I**T**F**T**L**H**LRR**RTLYYS**F**NL**IAP**VL**L**TMI**LV**IL**GF**T**V**S----PETC**EK**---V**GL**QISVS**LA**ICIFLTIMSELTPQTSEA-VPLL**G**V**FF**HTCNF**I**SVLATSFTVYVQSFHFR 162 EWRFAAIV**VD**---R**L**C-**LL**-A**F**SLLIVVVS**I**IIALRAPYLFA 515|

acr-12_Ce_17569167 SLTSER**H**SVL**Y**A-----------S**CC**GPE-K**Y**VD**I**T**Y**Y**F**G**LRR**KTLFFT**C**NL**ILP**CF**L**ISI**LT**TF**VF**Y**L**-------SD**HK**---I**TF**SISI**LVT**LTVFFLVLIDLMPPTSLV-IPMF**G**R**YL**ITT**M**I**L**VALST**V**VSVITVNFRFR 160 DWTFVAMV**LD**---R**L**F-**LI**-I**F**SVLNVGTV**F**IILESPSLYDY 548|

CHRNA1_Hs_4557457 ESRGWK**H**SVT**Y**S------------**CC**-PDTP**Y**LD**I**T**Y**H**F**V**MQR**LPLYFI**V**NV**IIP**CL**L**FSF**LT**GL**VF**Y**L**P----TDSG**EK**---M**TL**SISV**LLS**LTVFLLVIVELIPSTSSA-VPLI**G**K**YM**LFT**M**V**F**VIASI**I**ITVIVINTHHR 96 EWKYVAMV**MD**---H**I**L-**L**G-V**F**MLVCIIGT**L**AVFAGRLIELN 454|

ACh_Toma_113077 DYRGWK**H**WVY**Y**T------------**CC**-PDTP**Y**LD**I**T**Y**H**F**I**MQR**IPLYFV**V**NV**IIP**CL**L**FSF**LT**VL**VF**Y**L**P----TDSG**EK**---M**TL**SISV**LLS**LTVFLLVIVELIPSTSSA-VPLI**G**K**YM**LFT**M**I**F**VISSI**I**VTVVVINTHHR 96 EWKYVAMV**ID**---H**I**L-**LC**-V**F**MLICIIGT**V**SVFAGRLIELS 458|

1UV6_Lst_47169299 DVTQKK**N**SVT**Y**S------------**CC**-PE-A**Y**ED**V**E**V**S**L**N**FRK**KA---------**-**--------------------------------------------------------------------------------------------------------------------------------------- 205/

Consensus 90% ...............................h..h.h.h.hpR......h..hhP..h...hs..sh.h..........c....sh....hhs.......................s.hh...h.h.....h........................hp....h..b...h........h...........

Key Residues ..........#.............##.....#..............................................................................................................................................................
